# Anti-Annexin A5 antibodies and Annexin A5 resistance in antiphospholipid syndrome: A systematic review and meta-analysis

**DOI:** 10.1016/j.jtauto.2026.100373

**Published:** 2026-04-23

**Authors:** Laith Alamlih, Karima Becetti, Samar Al Emadi, Eman satti, Zahir M. Tag, Hiam Chemaitelly, Mohammad Abu-Tineh, Mohamad Abufaied, Sa'ad Laws, Munther Khamashta

**Affiliations:** a Department of Internal Medicine, Al-Quds University, Jerusalem, Palestine; b Division of Rheumatology, Department of Medicine, Hamad Medical Corporation, Qatar; c Weill Cornell Medicine, Doha, Qatar; d Infectious Disease Epidemiology Group, Weill Cornell Medicine-Qatar, Cornell University, Doha, Qatar; e Department of Population Health Sciences, Weill Cornell Medicine, Cornell University, New York, NY, USA; f Department of Internal Medicine, Reading Hospital-Tower Health, West Reading, PA, 19611, USA; g Princess of Wales Hospital, Bridgend, UK; h Emeritus Professor of Medicine, Dept of Women & Children's Health, King's College London, UK; i GSK Gulf Scientific Director, Immunology-Dubai, United Arab Emirates

**Keywords:** Antiphospholipid syndrome, Annexin A5 antibodies, Annexin A5 resistance, Non-criteria antiphospholipid antibodies, Obstetric APS, Thrombotic APS, Seronegative APS, Systemic lupus erythematosus, Meta-analysis, Biomarkers

## Abstract

**Objective:**

To provide an updated systematic review and meta-analysis of the estimated pooled of anti–Annexin A5 antibodies (AnxA5-Abs) and Annexin A5 resistance (A5R) across antiphospholipid syndrome (APS) phenotypes.

**Methods:**

PubMed, EMBASE, and the Cochrane Library were searched from inception to June 2018 and updated in August 2025. Eligible studies included adults tested for AnxA5-Abs (IgG/IgM) and/or A5R who fulfilled revised Sapporo/Sydney APS clinical criteria. Pooled prevalences were estimated using Freeman–Tukey random-effects models and stratified by phenotype, serologic status, and autoimmune disease. Univariable and multivariable meta-regression evaluated predictors of IgG AnxA5-Abs and A5R. The protocol was registered in PROSPERO (CRD42018099462).

**Results:**

Fifty-eight studies were included, comprising 6960 patients and 2417 controls. In obstetric APS cohorts, pooled IgG AnxA5-Abs prevalence was 21.2% (95% CI 7.4–39.2) versus 4.0% (1.7–7.1) in controls; thrombotic APS showed 10.3% (5.7–15.8). IgM AnxA5-Abs were less frequent and showed weaker associations. IgG AnxA5-Abs prevalence was higher in seropositive APS (27.1%) and seronegative APS (20.4%) than in mixed/unstratified APS (11.7%). Among systemic lupus erythematosus (SLE) patients with APS-related clinical manifestations, IgG AnxA5-Abs prevalence was 31.7% versus 16.3% in SLE controls without clinical APS manifestations. A5R prevalence was higher in obstetric (29.9%) and thrombotic APS (28.8%) than in controls (17.5%). Obstetric APS independently predicted higher IgG AnxA5-Abs prevalence.

**Conclusion:**

IgG AnxA5-Abs and A5R are enriched in Sapporo-defined obstetric APS, with weaker and less consistent associations in thrombosis-only APS. These findings support the biological plausibility of AnxA5 disruption in obstetric APS and identify AnxA5-related biomarkers as promising candidates for future validation. However, prospective external validation using standardized assays, pre-specified thresholds, and demonstration of incremental prognostic value are essential prerequisites before clinical risk stratification use.

## Highlights

• IgG anti–Annexin A5 antibodies and A5 resistance are enriched in Sapporo-defined obstetric APS versus controls.
• Associations are weaker and less consistent in thrombosis-only APS and non-Sapporo pregnancy loss cohorts.
• Findings support phenotype-specific relevance in obstetric APS, warranting prospective validation before clinical use.

## 1 Introduction

Antiphospholipid syndrome (APS) is a systemic autoimmune prothrombotic disorder defined by the occurrence of vascular thrombosis and/or pregnancy morbidity together with persistent laboratory evidence of lupus anticoagulant (LA), anticardiolipin (aCL), and/or anti–β_2_-glycoprotein I (anti-β_2_GPI) antibodies detected on two occasions at least 12 weeks apart [1]. Although this consensus framework has standardized APS classification, a subset of patients presents with the full clinical phenotype but remains negative for conventional serologic tests a condition referred to as *seronegative APS* (SNAPS) [2,3].

Over the past two decades, increasing attention has been directed toward non-criteria antiphospholipid antibodies (aPL) that may explain such SNAPS presentations. Among these, antibodies targeting phosphatidylethanolamine (aPE), phosphatidylserine/prothrombin complexes (aPS/PT), and Annexin A5 (AnxA5) have been repeatedly associated with thrombotic and obstetric complications, suggesting that conventional criteria do not capture the full spectrum of pathogenic aPL species [[4], [5], [6], [7]].

AnxA5 also known as placental anticoagulant protein-I—is a calcium-dependent phospholipid-binding protein abundantly expressed on endothelial and placental trophoblast surfaces [8,9]. By binding to anionic phospholipids such as phosphatidylserine, AnxA5 self-assembles into a two-dimensional crystalline lattice that forms a protective anticoagulant shield, preventing the assembly of prothrombinase and tenase complexes and thus limiting thrombin generation [10]. Disruption of this AnxA5 shield by aPL–β_2_GPI immune complexes exposes procoagulant phospholipids, promoting thrombosis and pregnancy loss [11,12].

Experimental and clinical studies have demonstrated that some aPL directly target AnxA5, leading to measurable *Annexin A5 resistance* (A5R), which is a functional phenotype reflecting reduced anticoagulant activity and impaired endothelial protection [12]. In these assays, patient plasma or purified IgG decreases the ability of exogenous AnxA5 to inhibit prothrombin generation, correlating with both thrombosis and pregnancy morbidity in APS cohorts [12].

Despite substantial experimental and clinical evidence, the true prevalence and diagnostic value of anti-Annexin A5 antibodies (AnxA5-Abs) and A5R remain uncertain. Reported frequencies vary widely across studies, reflecting differences in assay design, antigen sources, and patient populations. Some reports suggest that incorporating AnxA5-Abs or functional resistance assays may improve detection of clinically relevant APS particularly in SNAPS or obstetric subgroups—whereas others have found limited diagnostic gain compared with established aPL tests [4,5,12].

Given these discrepancies, a quantitative synthesis of available evidence is needed to determine the consistency, magnitude, and clinical implications of AnxA5-related abnormalities across APS phenotypes.

### 1.1 Objectives of the present study

This systematic review and meta-analysis aimed to [1]: estimate the pooled prevalence of AnxA5-Abs and A5R among patients meeting APS clinical criteria [2]; compare prevalence between seropositive APS, SNAPS, and healthy controls; and [3] explore associations of thrombotic and obstetric manifestations with prevalence of AnxA5-Abs and A5R. By integrating antibody-based and functional data, this study seeks to clarify the clinical relevance of AnxA5 biomarkers, identify APS subgroups in which AnxA5 disruption is most pronounced, and inform future updates to APS classification frameworks.

## 2 Methods

The review protocol was developed in accordance with the Cochrane Collaboration methodological guidelines [13]. Findings were reported according to the Preferred Reporting Items for Systematic Reviews and Meta-Analyses (PRISMA) 2020 statement [14]. The review was conducted from inception to June 2, 2018, with an updated literature search performed before manuscript submission (July 30, 2025). The review question was: *“What is the prevalence of* AnxA5-Abs *(IgG and IgM) and/or A5R among patients meeting the clinical criteria for APS?”*

### 2.1 Study outcomes and prioritization

#### 2.1.1 Primary objective

To estimate the prevalence of (AnxA5-Abs; IgG and IgM) positivity and/or A5R among patients fulfilling the clinical criteria of APS.

#### 2.1.2 Secondary objectives

1. To determine the prevalence of AnxA5-Abs and/or A5R in patients with APS fulfilling both clinical and laboratory criteria (i.e., at least one positive test for lupus anticoagulant, anticardiolipin, or anti-β_2_-glycoprotein I antibodies).
2. To estimate the prevalence of AnxA5-Abs and/or A5R among patients with pregnancy-related morbidity.
3. To estimate the prevalence of AnxA5-Abs and/or A5R among patients with venous or arterial thrombosis.
4. To determine the prevalence of AnxA5-Abs and/or A5R in patients fulfilling APS clinical criteria but testing negative for conventional aPL.
5. To evaluate the association between the prevalence of AnxA5-Abs and/or A5R and specific clinical manifestations of APS.

### 2.2 Definitions of APS classification and serologic categories

#### 2.2.1 APS clinical criteria used for study eligibility

Patients were considered to have APS if the original study reported classification according to the revised Sapporo/Sydney criteria (2006) or explicitly described patients as fulfilling APS diagnostic criteria.

Clinical manifestations included at least one of the following:

• **Vascular thrombosis:** Venous and/or arterial thrombosis confirmed by objective imaging or histopathology.
• **Pregnancy morbidity:**
  o One or more unexplained fetal deaths at ≥10 weeks' gestation, or
  oOne or more premature births before 34 weeks' gestation due to preeclampsia, eclampsia, or placental insufficiency, or
  oThree or more consecutive spontaneous pregnancy losses before 10 weeks' gestation unexplained by anatomic, hormonal, or chromosomal causes.

#### 2.2.2 LabSoratory criteria and serologic categorization

Laboratory APS classification requires positivity for at least one conventional antiphospholipid antibody (lupus anticoagulant, anticardiolipin IgG/IgM, or anti-β_2_-glycoprotein I IgG/IgM), typically confirmed on two occasions at least 12 weeks apart and, for aCL and anti-β_2_GPI, at medium or high titers.

For the purposes of this meta-analysis, studies were categorized according to the serologic profile as reported by the original investigators:

• **Seropositive APS:** Cohorts described as having at least one conventional aPL positive according to the study's definition.
• **Seronegative APS (SNAPS):** Cohorts fulfilling APS clinical criteria but reported as negative for conventional aPL.
• **Mixed serologic cohorts:** Studies including both seropositive and seronegative patients without stratified reporting.

Because many older studies predated the 2006 Sydney update, detailed reporting of persistence (≥12 weeks apart) and titer thresholds was inconsistently available. We therefore relied on each study's classification.

#### 2.2.3 Other pregnancy morbidity cohorts

Cohorts of women with unexplained pregnancy loss not fulfilling full APS classification criteria were included in the overall pooled analyses but examined separately in stratified analyses and meta-regression models to minimize misclassification bias.

### 2.3 Search strategy and study selection

A comprehensive search strategy was developed in accordance with the PROSPERO protocol (CRD42018099462) and Cochrane recommendations. PubMed, EMBASE (Ovid), and the Cochrane Library were searched from inception to June 2, 2018 and updated on July 30, 2025. This represents a single comprehensive update rather than a formal living systematic review. Additional sources included ProQuest Dissertations & Theses and the Web of Knowledge Conference Proceedings Citation Index. Searches combined MeSH/Emtree terms and free-text keywords for Annexin A5/Annexin V, antiphospholipid antibodies/syndrome, and APS-related clinical outcomes (thrombosis and pregnancy morbidity). Full search strings are provided in Supplementary Box S1. No language restrictions were applied.

All retrieved citations were imported into EndNote, where duplicates were removed using the Bramer method, and unique records were uploaded into Covidence for screening. Two reviewers independently screened titles/abstracts and classified records as “relevant,” “potentially relevant,” or “not relevant.” Disagreements were resolved by discussion or a third reviewer. Full texts of relevant or potentially relevant studies were assessed against predefined eligibility criteria, and reference lists of included articles were hand-searched for additional studies.

### 2.4 Eligibility criteria

#### 2.4.1 Inclusion

• Adults (≥18 years) tested for AnxA5-Abs (IgG/IgM) and/or A5R.
• Patients fulfilling APS clinical criteria according to the revised Sapporo/Sydney definitions (Supplementary Box S2).
• A predefined subgroup of women with unexplained pregnancy loss, analyzed separately due to its relevance to seronegative obstetric APS.

#### 2.4.2 Exclusion

Case reports, case series, editorials, commentaries, qualitative studies, narrative reviews, and animal or in vitro studies.

### 2.5 Data extraction and management

Data extraction was performed independently and in duplicate by two reviewers (L.I.A. and K.B.) using a standardized, pilot-tested data collection form developed in accordance with the Cochrane Handbook. Each reviewer extracted all variables separately, and results were subsequently cross-checked for accuracy. Discrepancies between reviewers were resolved through discussion and, when necessary, by consultation with a third reviewer (I.S.) or by contacting study authors for clarification.

The exact data extraction from each eligible study is provided in supplementary Box S3.

### 2.6 Risk of bias and precision assessment

Methodological quality was assessed using a pre-specified framework (PROSPERO: CRD42018099462) adapted from the Hoy et al. tool and Cochrane Handbook for prevalence studies. Five domains were evaluated:

1. **APS** classification **validity** (Sapporo/Sydney criteria vs. alternatives)
2. **Manifestation ascertainment** (e.g., imaging, obstetric documentation)
3. **Exclusion of alternative causes** (e.g., thrombophilia workup)
4. **Assay quality** (reporting of method, cut-off, reproducibility)
5. **Data completeness** (≥80% vs. <80%)

A study was classified as having low overall risk of bias (ROB) if at least three of the five assessed domains were rated as low ROB, and as having high overall ROB if at least three of the five domains were rated as high ROB. Studies that did not fulfill the criteria for either low or high ROB were classified as having unclear overall ROB. Studies with ≥100 samples were considered as having high precision due to reduced random error. The criteria used to assign low, high or unclear ROB for each domain are provided in Supplementary Box S4.

### 2.7 Data synthesis and analysis

Descriptive statistics were used to summarize the characteristics of included studies. ROB assessments across the five domains were visualized using traffic-light and weighted summary plots generated with the “robvis” package using R v.4.5.2 (R Foundation for Statistical Computing, Vienna, Austria). Stratified prevalence estimates were synthesized using medians and ranges.

Random-effects meta-analyses were conducted to estimate pooled mean prevalence of AnxA5 and A5R across population and biomarker subgroups, with corresponding 95% confidence intervals (CIs). A minimum of two measures was required to conduct a meta-analysis. Variances of prevalence measures were first stabilized using Freeman–Tukey double–arcsine square-root transformation [15], after which inverse-variance weights were applied. Weighted prevalence estimates were then pooled into a summary mean using a Restricted Maximum Likelihood model, selected for its methodological advantages over the DerSimonian–Laird approach [16].

Between-study heterogeneity was assessed using Cochran's Q statistic, with p < 0.1 indicating statistically significant heterogeneity. The I^2^ statistic quantified the proportion of variability attributable to true differences in prevalence rather than sampling error. Prediction intervals were calculated to characterize the expected distribution of true prevalence values around the pooled mean [17].

Linear random-effects meta-regression analyses were performed on logit-transformed proportions to investigate sources of between-study heterogeneity and identify associations with AnxA5-Abs and/or A5R prevalence [18]. Univariable models were initially fitted for a priori–defined variables—including APS phenotype and conventional aPL serologic status—as well as additional covariates identified during data extraction. Variables with p-value ≤0.2 in univariable analyses were included in the multivariable model, in which variables with p-value <0.05 were considered statistically significant. A subgroup meta-regression was also conducted for studies assessing AnxA5-Abs IgG prevalence. Odds ratios (ORs), adjusted ORs (aORs), and corresponding 95% CIs were reported.

The unit of analysis was the *measure*, defined as a single prevalence estimate for a specified biomarker within a defined study population or subgroup. A single study could contribute multiple measures if stratified data were reported (e.g., obstetric vs. thrombotic subgroups or separate IgG and IgM estimates). Each measure was treated as a distinct prevalence estimate for pooling purposes. Because measures originating from the same study may share methodological context, findings were interpreted with consideration of potential within-study correlation.

Meta-analyses and meta-regressions were conducted in R (v4.5.2) using the “meta” package [18]. To address potential within-study dependence arising from multiple estimates contributed by the same study, we performed a pre-specified sensitivity analysis using a one-study–one-effect approach. For each analysis, a single estimate per study was selected using a hierarchical framework prioritizing [1]: clinical phenotype (obstetric > thrombotic > mixed) [2], serologic status (seronegative > seropositive > mixed) [3], antibody isotype (IgG > IgM), and [4] sample size. Control groups were similarly restricted to one per study, prioritizing clinically relevant comparator populations. Sensitivity analyses were conducted for meta-regression models.

## 3 Results

### 3.1 Search Results and Study Characteristics

**Fig. 1:**
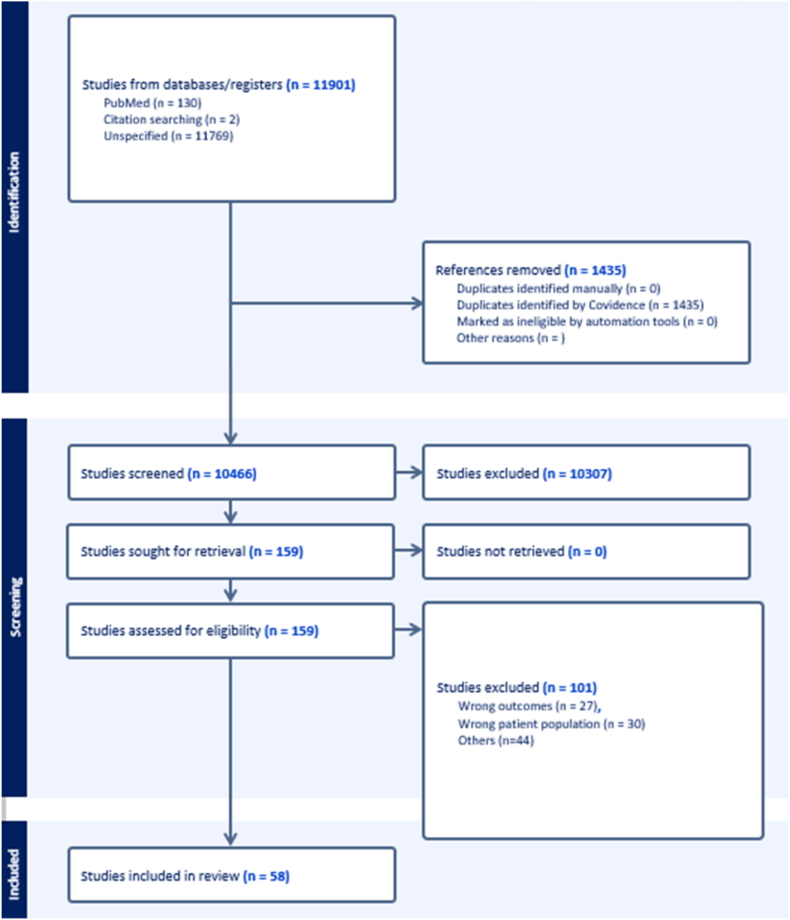
PRISMA 2020 Flow Diagram for Study Selection The flow diagram summarizes the identification, screening, eligibility assessment, and final inclusion of studies in the systematic review. A total of 11,901 records were identified, of which 1435 were removed as duplicates. After screening 10,466 records, 159 full texts were assessed for eligibility, and 58 studies met the inclusion criteria. Reasons for exclusion at the full-text stage included wrong outcomes (n = 27), wrong patient population (n = 30), and other reasons (n = 44).

Fig. 1 presents the PRISMA flow diagram illustrating the study selection process. A total of 11,901 citations were retrieved through the literature search. After removing 1435 duplicates, 10,466 unique records were screened by title and abstract. Of these, 10,307 were excluded as irrelevant, leaving 159 articles for full-text review. Following full-text assessment, 101 studies were excluded. Ultimately, 58 studies were included in the systematic review.

Table S1 summarizes the characteristics of the included studies. Publication years ranged from 1995 to 2024, with a median year of 2012. Collectively, these 58 studies contributed 169 stratified prevalence measures for inclusion in meta-analyses (Table S2). Most measures originated from cross-sectional studies and focused on primary APS populations. AnxA5-Abs IgG was the most frequently evaluated biomarker (47.9%), followed by AnxA5-Abs IgM (26.0%). Regarding conventional APS antibody status, measures predominantly involved mixed/unstratified serologic cohorts (39.1%), followed by seropositive cohorts (23.7%).

### 3.2 Risk of bias and precision assessments

**Fig. 2:**
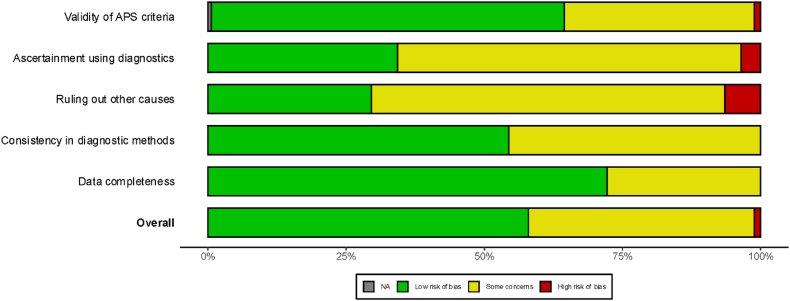
Summary plot of the risk of bias assessment for studies reporting AnxA5-Ab and A5R prevalence. Abbreviations: APS, antiphospholipid syndrome; Fig. 2. Summary of risk-of-bias assessment across included studies. *The plot displays the proportional distribution of judgments for each risk-of-bias domain, including validity of APS classification criteria, ascertainment using diagnostic methods, ruling out alternative causes, consistency in diagnostic procedures, and data completeness. Most domains demonstrated predominantly low risk of bias, with some studies showing areas of concern and a small proportion rated as high risk.* NA, not applicable.

Fig. S1 illustrates the study-specific ROB assessment across the five quality domains. Overall, the methodological quality of the included evidence was high, with few measures receiving high ROB ratings in any domain (Table S1 and Fig. 2).

Validity of APS case definitions was strong, with 63.9% of measures judged as low ROB and only 1.2% as high ROB. Diagnostic ascertainment and ruling out alternative causes were often insufficiently reported, leading to high proportions of unclear ROB (62.1% and 63.9%, respectively), yet high ROB ratings remained rare (3.6% and 6.5%). Consistency in diagnostic methods and data completeness were robust, with 54.4% and 72.2% of measures rated as low ROB and no high ROB in either domain. Most of the measures (74.0%) included 100 or more patients, suggesting high precision of prevalence estimates (Table S1).

### 3.3 AnxA5-Abs and A5R: pooled mean prevalence

Table 1 summarizes AnxA5-Abs and A5R prevalence estimates, presenting medians and ranges alongside pooled mean prevalence stratified by population group and biomarker type. Corresponding forest plots are shown in Fig. S2.

**Table 1:** Meta-analyses estimating the pooled mean AnxA5-Ab and A5R prevalence stratified by population group and biomarker type.

Populations	Measures	Sample		Prevalence (%)				Heterogeneity measures		
	Total N	Tested	Positive	Median	Range	Pooled mean	95% CI	Q^a^ (p-value)	I^2^^b^ (%, 95% CI)	Prediction interval^c^ (95% CI)
**Population type**										
Obstetric										
IgG	17	2023	236	5.8	2.9-54.5	**21.2**	7.4-39.2	616.9 (p < 0.001)	97.4 (96.7-98.0)	0.0-96.4
IgM	9	1671	44	3.3	2.5-7.0	**3.1**	1.4-5.3	25.5 (p = 0.001)	68.7 (37.2-84.4)	0.0-10.8
IgG/IgM/IgA	8	693	113	11.8	8.3-21.6	**14.2**	7.5-22.3	43.2 (p < 0.001)	83.8 (69.7-91.3)	0.0-43.7
A5R	4	358	103	34.7	23.0-43.2	**29.9**	13.1-49.9	46.9 (p < 0.001)	93.6 (86.8-96.9)	0.0-93.4
Unknown	2	59	5	8.4	8.2-8.6	**8.4**	2.2-17.4	0.0 (p = 0.956)	0.0 (--)	0.0-83.5
Thrombosis										
IgG	22	1577	134	9.7	3.2-25.0	**10.3**	5.7-15.8	157.9 (p < 0.001)	86.7 (81.2-90.6)	0.0-41.9
IgM	12	1100	38	2.6	1.0-6.7	**4.6**	0.4-12.1	86.5 (p < 0.001)	87.3 (79.6-92.1)	0.0-44.5
IgG/IgM/IgA	5	385	40	7.5	7.4-12.6	**10.2**	6.5-14.5	5.7 (p = 0.221)	30.1 (0.0-73.0)	2.8-21.0
A5R	5	672	153	18	14.9-52.0	**28.8**	13.2-47.4	68.9 (p < 0.001)	94.2 (89.3-96.9)	0.0-88.5
Unknown	1	122	1	--	--	**0.8**^d^	0.0-3.5	--	--	--
Mixed^e^										
IgG	54	4429	629	16.3	3.3-32.6	**18.3**	12.1-25.4	1195.4 (p < 0.001)	95.6 (94.8-96.2)	0.0-76.7
IgM	30	3417	157	4.1	1.8-9.9	**5.7**	2.9-9.3	228.0 (p < 0.001)	87.3 (83.0-90.5)	0.0-31.3
IgG/IgM/IgA	15	1253	256	12.6	7.9-23.2	**17.3**	9.8-26.2	212.2 (p < 0.001)	93.4 (90.7-95.3)	0.0-59.1
A5R	12	1256	399	44.7	17.2-52.5	**41.0**	24.5-58.5	318.2 (p < 0.001)	96.5 (95.2-97.5)	0.0-97.3
Unknown	4	217	6	4.4	0.6-8.2	**2.6**	0.0-8.6	8.1 (p = 0.043)	63.1 (0.0-87.6)	0.0-28.2
Control										
IgG	27	1542	77	4.2	0.0-12.5	**4.0**	1.7-7.1	125.0 (p < 0.001)	79.2 (70.3-85.4)	0.0-23.3
IgM	14	960	35	4.0	0.0-7.9	**3.1**	0.9-6.2	46.4 (p < 0.001)	72.0 (52.1-83.7)	0.0-17.2
IgG/IgM/IgA	3	184	77	16.0	12.2-35.9	**26.6**	4.1-58.1	32.1 (p < 0.001)	93.8 (85.2-97.4)	0.0-100.0
A5R	8	563	84	12.2	7.9-33.3	**17.5**	7.8-29.7	54.4 (p < 0.001)	87.1 (76.8-92.9)	0.0-63.8
Unknown	2	100	3	2.2	1.1-3.3	**1.9**	0.0-8.1	1.7 (p = 0.191)	41.4 (--)	0.0-96.3
**APS status**										
Seropositive										
IgG	19	1018	321	30.4	16.9-37.9	**27.1**	16.5-39.0	248.9 (p < 0.001)	92.8 (90.1-94.7)	0.0-81.9
IgM	13	767	91	7.5	5.6-16.6	**10.6**	4.9-18.0	72.6 (p < 0.001)	83.5 (73.1-89.8)	0.0-44.3
IgG/IgM/IgA	1	115	77	--	--	**67.0**^d^	58.1-75.3	--	--	--
A5R	7	533	270	52.0	44.7-72.0	**58.4**	38.3-77.3	105.2 (p < 0.001)	94.3 (90.6-96.5)	2.7-100.0
Unknown	--	--	--	--	--	**--**	--	--	--	--
Control-seropositive										
IgG	--	--	--	--	--	**--**	--	--	--	--
IgM	--	--	--	--	--	**--**	--	--	--	--
IgG/IgM/IgA	--	--	--	--	--	**--**	--	--	--	--
A5R	1	16	6	--	--	**37.5**^d^	15.1-62.8	--	--	--
Unknown	--	--	--	--	--	**--**	--	--	--	--
Seronegative										
IgG	8	252	83	7.6	2.5-35.7	**20.4**	1.3-50.9	205.7 (p < 0.001)	96.6 (94.9-97.7)	0.0-100.0
IgM	3	75	9	3.3	1.7-28.3	**11.3**	0.0-51.4	22.7 (p < 0.001)	91.2 (77.2-96.6)	0.0-100.0
IgG/IgM/IgA	2	117	41	34.8	30.6-39.1	**34.7**	19.1-52.0	3.7 (p = 0.055)	72.7 (0.0-93.9)	0.0-100.0
A5R	3	503	68	14.9	11.6-14.9	**13.2**	9.7-17.1	3.2 (p = 0.199)	38.1 (0.0-80.6)	4.0-26.4
Unknown	1	36	0	--	--	**0.0**^d^	0.0-4.7	--	--	--
Mixed										
IgG	30	3305	232	5.4	2.9-28.8	**11.7**	5.9-18.8	366.9 (p < 0.001)	92.1 (89.8-93.9)	0.0-61.2
IgM	16	2705	60	2.3	1.3-3.6	**2.0**	1.0-3.3	43.7 (p < 0.001)	65.7 (41.7-79.8)	0.0-7.7
IgG/IgM/IgA	14	1193	214	11.4	7.7-17.2	**14.5**	8.4-21.7	145.5 (p < 0.001)	91.1 (86.8-94.0)	0.0-47.5
A5R	3	366	79	18.0	15.2-33.3	**24.6**	7.2-47.8	32.9 (p < 0.001)	93.9 (85.6-97.4)	0.0-100.0
Unknown	3	181	6	8.0	4.4-8.4	**4.1**	0.0-12.9	7.0 (p = 0.030)	71.6 (3.5-91.6)	0.0-52.3
Control										
IgG	24	1396	70	4.1	0.0-11.2	**3.9**	1.6-6.9	106.0 (p < 0.001)	78.3 (68.2-85.2)	0.0-21.6
IgM	12	830	32	5.4	0.0-8.2	**3.6**	1.0-7.4	44.7 (p < 0.001)	75.4 (56.8-86.0)	0.0-20.6
IgG/IgM/IgA	1	12	1	--	--	**8.3**^d^	0.0-32.4	--	--	--
A5R^f^	7	417	66	12.0	7.9-34.7	**18.5**	7.2-33.1	54.1 (p < 0.001)	88.9 (79.6-94.0)	0.0-71.4
Unknown	2	100	3	2.2	1.1-3.3	**1.9**	0.0-8.1	1.7 (p = 0.191)	41.4 (--)	0.0-96.3
**Autoimmune disorder**										
SLE										
IgG	8	195	61	31.7	29.1-38.6	**31.7**	19.8-44.7	20.2 (p = 0.005)	65.3 (26.2-83.7)	2.7-71.4
IgM	--	--	--	--	--	**--**	--	--	--	--
IgG/IgM/IgA	--	--	--	--	--	**--**	--	--	--	--
A5R	2	160	36	54.0	36.0-72.0	**52.0**	0.0-100.0	22.5 (p < 0.001)	95.5 (87.0-98.5)	0.0-100.0
Unknown	--	--	--	--	--	**--**	--	--	--	--
Mixed										
IgG	31	2365	398	12.5	3.2-30.2	**18.2**	10.1-27.9	707.2 (p < 0.001)	95.8 (94.8-96.6)	0.0-79.7
IgM	18	1839	110	3.2	2.0-9.4	**6.0**	2.4-10.8	133.0 (p < 0.001)	87.2 (81.3-91.3)	0.0-34.3
IgG/IgM/IgA	13	1162	252	13.5	8.3-26.3	**19.8**	11.5-29.6	190.7 (p < 0.001)	93.7 (90.9-95.6)	0.0-62.2
A5R	8	976	308	38.0	14.9-49.9	**37.9**	17.7-60.4	276.5 (p < 0.001)	97.5 (96.4-98.2)	0.0-99.4
Unknown	4	217	6	4.4	0.6-8.2	**2.6**	0.0-8.6	8.1 (p = 0.043)	63.1 (0.0-87.6)	0.0-28.2
No autoimmune disease										
IgG	15	1869	170	4.4	1.3-27.2	**12.7**	3.5-25.9	351.3 (p < 0.001)	96.0 (94.6-97.0)	0.0-77.5
IgM	12	1578	47	6.0	1.5-9.7	**5.5**	1.4-11.5	76.3 (p < 0.001)	85.6 (76.5-91.2)	0.0-34.6
IgG/IgM/IgA	2	91	4	4.7	3.3-6.1	**4.1**	0.2-11.0	1.5 (p = 0.225)	32.2 (--)	0.0-91.7
A5R	2	120	55	46.7	44.1-49.4	**46.0**	35.9-56.3	1.3 (p = 0.257)	22.3 (--)	0.0-100.0
Unknown	--	--	--	--	--	**--**	--	--	--	--
Control^g^										
IgG	27	1542	77	4.2	0.0-12.5	**4.0**	1.7-7.1	125.0 (p < 0.001)	79.2 (70.3-85.4)	0.0-23.3
IgM	14	960	35	4.0	0.0-7.9	**3.1**	0.9-6.2	46.4 (p < 0.001)	72.0 (52.1-83.7)	0.0-17.2
IgG/IgM/IgA	3	184	77	16.0	12.2-35.9	**26.6**	4.1-58.1	32.1 (p < 0.001)	93.8 (85.2-97.4)	0.0-100.0
A5R	8	563	84	12.2	7.9-33.3	**17.5**	7.8-29.7	54.4 (p < 0.001)	87.1 (76.8-92.9)	0.0-63.8
Unknown	2	100	3	2.2	1.1-3.3	**1.9**	0.0-8.1	1.7 (p = 0.191)	41.4 (--)	0.0-96.3
Control-RA										
IgG	2	20	3	15.0	12.5-17.5	**14.7**	1.4-35.2	0.3 (p = 0.577)	0.0 (--)	0.0-100.0
IgM	--	--	--	--	--	**--**	--	--	--	--
IgG/IgM/IgA	--	--	--	--	--	**--**	--	--	--	--
A5R	--	--	--	--	--	**--**	--	--	--	--
Unknown	--	--	--	--	--	**--**	--	--	--	--
Control-SLE										
IgG	3	209	35	17.6	16.3-19.9	**16.3**	11.4-21.8	0.8 (p = 0.683)	0.0 (0.0-89.6)	6.5-29.1
IgM	--	--	--	--	--	**--**	--	--	--	--
IgG/IgM/IgA	--	--	--	--	--	**--**	--	--	--	--
A5R	3	232	53	31.9	22.1-41.6	**29.8**	9.9-54.6	27.0 (p < 0.001)	92.6 (81.6-97.0)	0.0-100.0
Unknown	--	--	--	--	--	**--**	--	--	--	--
Control-no autoimmune disease										
IgG	10	506	12	0.0	0.0-3.9	**1.2**	0.0-3.2	13.7 (p = 0.132)	34.5 (0.0-68.8)	0.0-7.4
IgM	6	257	8	1.7	0.0-7.5	**1.9**	0.0-5.9	9.9 (p = 0.077)	49.7 (0.0-80.0)	0.0-15.2
IgG/IgM/IgA	1	50	8	--	--	**16.0**^d^	7.0-27.6	--	--	--
A5R	2	65	8	20.8	12.4-29.1	**16.4**	0.0-58.4	9.7 (p = 0.002)	89.7 (61.7-97.2)	0.0-100.0
Unknown	--	--	--	--	--	**--**	--	--	--	--
Control-RA and SLE										
IgG	5	229	38	17.6	15.0-20.0	**15.5**	10.7-20.8	1.1 (p = 0.898)	0.0 (0.0-79.2)	8.9-23.2
IgM	--	--	--	--	--	**--**	--	--	--	--
IgG/IgM/IgA	1	122	68	--	--	**55.7**^d^	46.8-64.5	--	--	--
A5R	3	232	53	31.9	22.1-41.6	**29.8**	9.9-54.6	27.0 (p < 0.001)	92.6 (81.6-97.0)	0.0-100.0
Unknown	--	--	--	--	--	--	--	--	--	--

**Measures vs. Samples:** A single study may contribute multiple “measures” if stratified data are reported (e.g., obstetric vs. thrombotic subgroups, or separate IgG and IgM estimates). Each measure represents a distinct prevalence estimate within a defined study subgroup and was treated as a separate pooled data point in the meta-analysis. “Total N Measures” indicates the number of pooled prevalence estimates, whereas “Sample Tested” reflects the cumulative number of participants across contributing measures.

Abbreviations: APS, antiphospholipid syndrome; CI, confidence interval; IgA, immunoglobulin A; IgG, immunoglobulin G; IgM, immunoglobulin M; RA, rheumatoid arthritis; ROB, risk of bias; SLE, systemic lupus erythematosus.

a Q: Cochran's Q statistic, a measure used to assess the presence of heterogeneity in effect sizes (here, prevalence) across studies.

b I^2^: A measure indicating the proportion of total variation across studies attributable to heterogeneity in effect sizes (here, prevalence) rather than chance.

c Prediction interval: The estimated 95% interval representing the distribution of true effect sizes (here, prevalence) around the estimated mean.

d Point estimate derived from a single study.

e Includes all obstetric, thrombosis, and mixed populations.

f Includes control seropositive groups.

g Includes all controls (with or without SLE, with or without RA).

In obstetric APS cohorts, pooled IgG anti–Annexin A5 antibody prevalence was 21.2% (95% CI 7.4–39.2%), substantially higher than the 4.0% (95% CI 1.7–7.1%) observed in controls. In contrast, IgM AnxA5-Abs were uncommon (3.1% [95% CI 1.4–5.3%]) and comparable to controls (3.1% [95% CI 0.9–6.2%]).

Among thrombotic APS cohorts, pooled IgG AnxA5-Abs prevalence was 10.3% (95% CI 5.7–15.8%), while IgM prevalence was 4.6% (95% CI 0.4–12.1%). Both estimates exceeded corresponding control values, although effect sizes were smaller than in obstetric APS.

For A5R, pooled prevalence was elevated in obstetric APS (29.9% [95% CI 13.1–49.9%]) and thrombotic APS (28.8% [95% CI 13.2–47.4%]) compared with controls (17.5% [95% CI 7.8–29.7%]).

Stratification by conventional aPL status showed pooled IgG AnxA5-Abs prevalence of 27.1% (95% CI 16.5–39.0%) in seropositive APS, 20.4% (95% CI 1.3–50.9%) in seronegative APS, and 11.7% (95% CI 5.9–18.8%) in mixed or unstratified cohorts. Corresponding IgM prevalences were 10.6% (95% CI 4.9–18.0%), 11.3% (95% CI 0.0–51.4%), and 2.0% (95% CI 1.0–3.3%), respectively. For A5R, pooled prevalence was 58.4% (95% CI 38.3–77.3%) in seropositive APS, 13.2% (95% CI 9.7–17.1%) in seronegative APS, and 24.6% (95% CI 7.2–47.8%) in mixed or unstratified cohorts.

Among patients with systemic lupus erythematosus (SLE) and APS-related clinical manifestations, pooled IgG AnxA5-Abs prevalence was 31.7% (95% CI 19.8–44.7%), compared with 16.3% (95% CI 11.4–21.8%) in SLE controls without clinical APS features. A5R prevalence showed a similar pattern (52.0% [95% CI 0.0–100%] vs 29.8% [95% CI 9.9–54.6%]).

In studies reporting IgG AnxA5 by conventional aPL status (Table 1), crude positivity was 31.5% (321/1018) in seropositive APS cohorts and 32.9% (83/252) in seronegative APS cohorts. In contrast, mixed or unstratified cohorts demonstrated lower crude positivity (7.0% [232/3305]), consistent with dilution when conventional serologic status is not separated. Across contributing datasets, 83 of 636 IgG AnxA5-positive observations (13.0%) arose from seronegative APS cohorts.

As shown in Table S3, pooled IgG AnxA5-Abs prevalence followed a graded distribution: highest in stillbirth, followed by Sapporo-classified obstetric APS, then recurrent pregnancy loss not meeting Sapporo criteria, with substantially lower prevalence in thrombotic APS. A5R demonstrated a similar descending gradient. Within thrombotic APS, IgG AnxA5-Abs positivity was highest among patients with mixed arterial and venous events.

Most pooled analyses demonstrated statistically significant heterogeneity (p < 0.1), with I^2^ values frequently exceeding 75%, indicating substantial between-study variability. Wide prediction intervals further suggested that true prevalence may vary considerably across clinical settings. Potential sources of heterogeneity were explored through meta-regression analyses described below.

### 3.4 Associations with AnxA5-Abs prevalence

Table 2 summarizes the meta-regression analyses for AnxA5-Abs prevalence. In univariable analyses, population type, biomarker type, autoimmune disorder, APS status, and sample size had a p-value of ≤0.20 and were included in the multivariable models. Two models were fitted: Model 1 excluded autoimmune disorder due to collinearity, whereas Model 2 retained it to evaluate its independent effect.

**Table 2:** Results of meta-regressions for the association with AnxA5-Abs prevalence.

Characteristics	Measures	Samples	Univariable analysis			Multivariable analysis			
						Model 1^a^		Model 2^a^	
	N	Tested	OR (95% CI)	p-value	Global p-value^b^	aOR (95% CI)	p-value^c^	aOR (95% CI)	p-value^c^
Population type									
Control	46	2786	1.00		<0.001	1.00		1.00	
Sapporo or still birth	35	1963	5.11 (2.64-9.89)	<0.001		1.90 (0.56-6.43)	0.305	4.68 (1.22-17.91)	0.024
Other pregnancies (not Sapporo)	28	4169	1.17 (0.59-2.30)	0.657		0.69 (0.24-2.02)	0.498	2.34 (0.61-9.03)	0.216
Thrombosis	40	3184	1.22 (0.64-2.33)	0.538		0.64 (0.22-1.87)	0.419	1.86 (0.52-6.63)	0.338
Biomarker									
IgM	44	4377	1.00		0.001	1.00		1.00	
IgG	81	5971	2.55 (1.44-4.52)	0.001		2.47 (1.54-3.97)	<0.001	2.08 (1.30-3.34)	0.002
IgG/IgM/IgA	18	1437	3.38 (1.52-7.51)	0.003		4.22 (2.15-8.31)	<0.001	4.29 (2.23-8.29)	<0.001
Unknown	6	317	0.60 (0.14-2.63)	0.497		0.66 (0.19-2.36)	0.523	0.78 (0.22-2.70)	0.690
Autoimmune disorder									
Control-mixed^d^	43	2577	1.00		0.003	--	--	1.00	
Control-SLE	3	209	3.49 (0.66-18.50)	0.141		--	--	3.00 (0.83-10.89)	0.094
Mixed^d^	95	9121	1.99 (1.08-3.65)	0.027		--	--	0.27 (0.11-0.67)	0.005
SLE	8	195	7.67 (2.47-23.84)	<0.001		--	--	Omitted	
Autoimmune disorder									
Control-mixed^e^	29	1973	1.00		0.018	--	--	--	--
Control-no autoimmune disease	17	813	0.44 (0.14-1.33)	0.146		--	--	--	--
Mixed^e^	74	5778	1.81 (0.91-3.60)	0.091		--	--	--	--
No autoimmune disease	29	3538	1.02 (0.45-2.36)	0.954		--	--	--	--
APS status									
Control	39	2338	1.00		<0.001	1.00		1.00	
Seronegative	14	480	4.96 (1.86-13.19)	0.001		2.65 (0.64-10.98)	0.179	4.36 (1.06-17.85)	0.041
Seropositive	33	1900	4.56 (2.28-9.15)	<0.001		2.98 (0.82-10.86)	0.098	4.80 (1.32-17.37)	0.017
Mixed	63	7384	1.36 (0.73-2.51)	0.331		1.67 (0.56-4.97)	0.360	1.86 (0.64-5.45)	0.256
Year of data collection (median)	149	12,082	1.00 (0.97-1.04)	0.842	0.842				
Validity of APS criteria									
Low ROB	100	8756	1.00		0.950	--	--	--	--
High ROB	2	66	0.72 (0.08-6.39)	0.769		--	--	--	--
Unclear	46	3132	1.03 (0.58-1.82)	0.916		--	--	--	--
Ascertainment using diagnostics									
Low ROB	56	6442	1.00		0.413	--	--	--	--
High ROB	6	162	2.21 (0.62-7.90)	0.221		--	--	--	--
Unclear	87	5498	0.95 (0.55-1.63)	0.848		--	--	--	--
Ruling out other causes									
Low ROB	48	4481	1.00		0.584	--	--	--	--
High ROB	11	1666	0.68 (0.25-1.85)	0.444		--	--	--	--
Unclear	90	5955	1.11 (0.63-1.97)	0.717		--	--	--	--
Consistency in diagnostic methods									
Low ROB	74	7792	1.00		0.297	--	--	--	--
Unclear	75	4310	0.76 (0.45-1.28)	0.297		--	--	--	--
Data completeness									
Low ROB	112	9202	1.00		0.365	--	--	--	--
Unclear	37	2900	1.31 (0.73-2.35)	0.365		--	--	--	--
Sample size									
<100	113	4615	1.00		<0.001	1.00		1.00	
≥100	36	7487	0.37 (0.22-0.64)	<0.001		0.47 (0.29-0.75)	0.002	0.54 (0.34-0.86)	0.010

Abbreviations: aOR, adjusted odds ratio; APS, antiphospholipid syndrome; CI, confidence interval; IgA, immunoglobulin A; IgG, immunoglobulin G; IgM, immunoglobulin M; OR, odds ratio; RA, rheumatoid arthritis; ROB, risk of bias; SLE, systemic lupus erythematosus.

a Two multivariable logistic regression models were constructed. The first model excluded autoimmune disorder due to collinearity, whereas the second model retained this variable.

b Covariates with p-value ≤0.2 in the univariable analysis were included in the multivariable analysis.

c Covariates with p-value <0.05 in the multivariable analysis were considered as showing statistically significant evidence for an association with AnxA5-Abs prevalence.

d Mixed population includes controls and cases with no autoimmune disease, RA, and mixed status.

e Mixed population includes controls and cases with SLE, RA, and mixed status.

In multivariable meta-regressions, several factors were independently associated with higher AnxA5-Abs prevalence, with broadly consistent estimates across both models. Compared with controls, Sapporo-defined pregnancy loss and stillbirth cohorts had significantly higher odds of AnxA5-Abs positivity (aOR 4.68, 95% CI 1.22–17.91). Biomarker type was also a strong determinant, with IgG-based assays associated with higher prevalence than IgM (aOR 2.08, 95% CI 1.30–3.34). APS serologic profile was an important predictor: both seronegative APS (aOR 4.36, 95% CI 1.06–17.85) and seropositive APS (aOR 4.80, 95% CI 1.32–17.37) showed elevated odds relative to controls.

Larger studies (≥100 participants) demonstrated lower odds of AnxA5-Abs positivity (aOR 0.54, 95% CI 0.34–0.86), indicating a small-study effect. In Model 2, mixed autoimmune cohorts showed reduced odds of AnxA5-Abs (aOR 0.27, 95% CI 0.11–0.67) compared to controls. No risk-of-bias domain was significantly associated with prevalence. The similarity in direction and magnitude of associations across both models supports the robustness of these findings.

Results from the analyses restricted to IgG-based AnxA5-Abs assessments (Table 3) were consistent with the main analysis. Sapporo-defined stillbirth cohorts showed markedly higher odds of AnxA5-Abs IgG positivity (Model 1 aOR 11.41, 95% CI 1.55–84.07; Model 2 aOR 13.05, 95% CI 1.77–96.06), whereas neither autoimmune disorder nor APS serologic status showed significant associations with higher prevalence.

**Table 3:** Results of meta-regressions for the association with AnxA5-Abs prevalence as assessed using IgG.

Characteristics	Measures	Samples	Univariable analysis			Multivariable analysis			
						Model 1^a^		Model 2^a^	
	N	Tested	OR (95% CI)	p-value	Global p-value^b^	aOR (95% CI)	p-value^c^	aOR (95% CI)	p-value^c^
Population type									
Control	27	1542	1.00		<0.001	1.00		1.00	
Sapporo or still birth	21	1042	9.46 (3.97-22.53)	<0.001		11.41 (1.55-84.07)	0.017	13.05 (1.77-96.06)	0.012
Other pregnancies (not Sapporo)	11	1810	1.52 (0.56-4.15)	0.412		2.80 (0.41-19.16)	0.293	4.24 (0.55-32.66)	0.166
Thrombosis	22	1577	1.66 (0.71-3.88)	0.246		2.41 (0.39-15.04)	0.346	3.14 (0.48-20.57)	0.232
Autoimmune disorder									
Control-mixed^d^	24	1333	1.00		0.004	--	--	1.00	
Control-SLE	3	209	4.35 (0.68-27.71)	0.119		--	--	3.89 (0.93-16.26)	0.062
Mixed^d^	46	4234	3.50 (1.43-8.56)	0.006		--	--	0.49 (0.16-1.45)	0.197
SLE	8	195	9.50 (2.56-35.23)	0.001		--	--	Omitted	
Autoimmune disorder									
Control-mixed^e^	17	1036	1.00		0.008	--	--	--	--
Control-no autoimmune disease	10	506	0.27 (0.06-1.34)	0.110		--	--	--	--
Mixed^e^	39	2560	2.67 (1.03-6.96)	0.044		--	--	--	--
No autoimmune disease	15	1869	1.46 (0.45-4.76)	0.531		--	--	--	--
APS status									
Control	24	1396	1.00		<0.001	1.00		1.00	
Seronegative	8	252	5.25 (1.35-20.41)	0.017		0.66 (0.07-6.22)	0.720	1.55 (0.15-16.18)	0.716
Seropositive	19	1018	7.21 (2.77-18.81)	<0.001		0.63 (0.07-5.30)	0.669	1.47 (0.15-13.99)	0.737
Mixed	30	3305	1.79 (0.75-4.26)	0.191		0.60 (0.09-3.92)	0.594	1.01 (0.15-6.81)	0.989
Year of data collection (median)	81	5971	0.99 (0.94-1.04)	0.705	0.705	--	--	--	--
Validity of APS criteria									
Low ROB	58	4607	1.00		0.681	--	--	--	--
Unclear	22	1216	1.20 (0.50-2.85)	0.681		--	--	--	--
Ascertainment using diagnostics									
Low ROB	33	3379	1.00		0.783	--	--	--	--
High ROB	2	48	1.97 (0.19-20.47)	0.571		--	--	--	--
Unclear	46	2544	0.89 (0.40-1.97)	0.770		--	--	--	--
Ruling out other causes									
Low ROB	30	2603	1.00		0.860	--	--	--	--
High ROB	4	704	1.37 (0.24-7.74)	0.720		--	--	--	--
Unclear	47	2664	0.88 (0.39-2.02)	0.768		--	--	--	--
Consistency in diagnostic methods									
Low ROB	44	4138	1.00		0.488	--	--	--	--
Unclear	37	1833	0.76 (0.35-1.65)	0.488		--	--	--	--
Data completeness									
Low ROB	65	4896	1.00		0.069	1.00		1.00	
Unclear	16	1075	2.35 (0.94-5.90)	0.069		2.35 (1.13-4.89)	0.022	1.88 (0.89-3.96)	0.097
Sample size									
<100	65	2537	1.00		0.001	1.00		1.00	
≥100	16	3434	0.24 (0.11-0.55)	0.001		0.27 (0.13-0.54)	<0.001	0.31 (0.15-0.65)	0.002

Abbreviations: aOR, adjusted odds ratio; APS, antiphospholipid syndrome; CI, confidence interval; IgG, immunoglobulin G; RA, rheumatoid arthritis; ROB, risk of bias; SLE, systemic lupus erythematosus.

a Two multivariable logistic regression models were constructed. The first model excluded autoimmune disorder due to collinearity, whereas the second model retained this variable.

b Covariates with p-value ≤0.2 in the univariable analysis were included in the multivariable analysis.

c Covariates with p-value <0.05 in the multivariable analysis were considered as showing statistically significant evidence for an association with AnxA5-Abs prevalence.

d Mixed population includes controls and cases with RA, mixed status, and no autoimmune disease.

e Mixed population includes controls and cases with RA, SLE, and mixed status.

### 3.5 Associations with AnxA5 resistance prevalence

Table 4 presents the meta-regression analyses for A5R. In univariable screening, population type, autoimmune disorder, APS status, data completeness, and sample size met inclusion criteria for multivariable modeling (p ≤ 0.20). In the multivariable analyses, no factor demonstrated an independent association with A5R, and effect estimates were broadly consistent across both Model 1 (excluding autoimmune disease to reduce collinearity) and Model 2 (retaining it).

**Table 4:** Results of meta-regressions for the association with A5R prevalence.

Characteristics	Measures	Samples	Univariable analysis			Multivariable analysis			
						Model 1∗		Model 2∗	
	N	Tested	OR (95% CI)	p-value	Global p-value^a^	aOR (95% CI)	p-value^b^	aOR (95% CI)	p-value^b^
Population type									
Control	8	563	1.00		0.037	1.00		1.00	
Sapporo or still birth	2	136	15.08 (2.22-102.41)	0.006		2.68 (0.09-82.18)	0.573	15.30 (0.17-1371.11)	0.234
Other pregnancies (not Sapporo)	5	448	3.18 (0.79-12.81)	0.104		1.81 (0.13-25.29)	0.660	11.69 (0.19-704.89)	0.240
Thrombosis	5	672	1.94 (0.50-7.50)	0.339		1.78 (0.12-25.86)	0.674	8.84 (0.20-382.19)	0.257
Autoimmune disorder									
Control-mixed^c^	5	331	1.00		0.128	--	--	1.00	
Control-SLE	3	232	3.33 (0.51-21.87)	0.211		--	--	3.99 (0.43-37.11)	0.225
Mixed^c^	10	1096	5.13 (1.23-21.42)	0.025		--	--	0.46 (0.03-7.01)	0.576
SLE	2	160	7.26 (0.73-72.69)	0.092		--	--	Omitted	
Autoimmune disorder									
Control-mixed^d^	6	498	1.00		0.276	--	--	--	--
Control-no autoimmune disease	2	65	0.85 (0.09-8.41)	0.887		--	--	--	--
Mixed^d^	10	1136	3.07 (0.77-12.26)	0.111		--	--	--	--
No autoimmune disease	2	120	4.28 (0.49-37.10)	0.187		--	--	--	--
APS status									
Control	7	417	1.00		0.002	1.00		1.00	
Seronegative	3	503	0.68 (0.16-2.89)	0.598		1.07 (0.04-31.32)	0.971	0.61 (0.01-39.34)	0.818
Seropositive	7	533	6.79 (2.13-21.66)	0.001		4.42 (0.22-87.76)	0.330	2.26 (0.06-82.73)	0.657
Mixed	3	366	1.46 (0.34-6.21)	0.608		1.88 (0.16-21.44)	0.612	0.67 (0.03-14.01)	0.799
Year of data collection (median)	20	1819	0.99 (0.79-1.25)	0.960	0.960	--	--	--	--
Validity of APS criteria									
Low ROB	8	410	1.00		0.142	1.00		1.00	
Unclear	12	1409	0.41 (0.13-1.35)	0.142		2.59 (0.38-17.56)	0.329	0.93 (0.06-14.18)	0.960
Ascertainment using diagnostics									
Low ROB	2	120	1.00		0.910	--	--	--	--
Unclear	18	1699	1.13 (0.14-9.01)	0.910		--	--	--	--
Ruling out other causes									
Low ROB	2	120	1.00		0.910	--	--	--	--
Unclear	18	1699	1.13 (0.14-9.01)	0.910		--	--	--	--
Consistency in diagnostic methods									
Low ROB	18	1699	1.00		0.910	--	--	--	--
Unclear	2	120	0.89 (0.11-7.09)	0.910		--	--	--	--
Data completeness									
Low ROB	10	467	1.00		0.022	1.00		1.00	
Unclear	10	1352	0.28 (0.09-0.84)	0.022		0.18 (0.02-1.83)	0.148	0.62 (0.03-15.16)	0.770
Sample size									
<100	12	619	1.00		0.033	1.00		1.00	
≥100	8	1200	0.30 (0.10-0.91)	0.033		0.65 (0.10-4.40)	0.660	0.49 (0.06-3.88)	0.503

Abbreviations: aOR, adjusted odds ratio; APS, antiphospholipid syndrome; CI, confidence interval; OR, odds ratio; RA, rheumatoid arthritis; ROB, risk of bias; SLE, systemic lupus erythematosus.

∗∗Two multivariable logistic regression models were constructed. The first model excluded autoimmune disorder due to collinearity, whereas the second model retained this variable.

a Covariates with p-value ≤0.2 in the univariable analysis were included in the multivariable analysis.

b Covariates with p-value <0.05 in the multivariable analysis were considered as showing statistically significant evidence for an association with A5R prevalence.

c Mixed population includes controls and cases with RA, mixed status, and no autoimmune disease.

d Mixed population includes controls and cases with RA, SLE, and mixed status.

Both models showed suggestive but non-significant positive trends. Sapporo-defined stillbirth cohorts demonstrated the largest upward signals, with Model 1 showing an adjusted odds ratio of 2.68 (p = 0.573) and Model 2 an aOR of 15.30 (p = 0.234), although both estimates were accompanied by extremely wide confidence intervals due to very limited contributing data. Seropositive APS also showed a directionally positive association in both models—Model 1 aOR 4.42 (p = 0.330) and Model 2 aOR 2.26 (p = 0.657)—but without statistical significance. Other pregnancy-related cohorts showed similar patterns, with upward trends that did not reach significance and were characterized by considerable imprecision.

Taken together, multivariable analyses suggest that while certain APS phenotypes may have higher odds of A5R, the available evidence remains inconclusive, driven largely by sparse data, heterogeneous assay methods, and wide confidence intervals that limit the precision and interpretability of these estimates.

Sensitivity analyses using a one-study–one-effect approach yielded results consistent with the primary analyses. The direction and magnitude of associations remained unchanged, with IgG AnxA5 remaining enriched in obstetric APS compared with controls. As expected, confidence intervals were wider due to reduced sample size, but no meaningful changes in statistical significance or interpretation were observed. These findings support the robustness of the primary results and suggest that within-study dependence did not materially influence the conclusions (Tables S4–6).

## 4 Discussion

This meta-analysis provides the first comprehensive synthesis of both AnxA5-Abs and A5R across APS phenotypes. Our findings demonstrate that IgG AnxA5-Abs are significantly enriched in Sapporo-defined obstetric APS, with pooled prevalence of 21.2% (95% CI 7.4–39.2%) compared with 4.0% (1.7–7.1%) in controls. In contrast, IgM AnxA5-Abs were uncommon and did not exceed control rates [19]. A5R which is a functional marker reflecting disruption of Annexin A5's anticoagulant shield was elevated in both obstetric (29.9%, 95% CI 13.1–49.9%) and thrombotic APS (28.8%, 13.2–47.4%) compared with controls (17.5%, 7.8–29.7%).

Both biomarkers showed enrichment in seronegative APS cohorts (IgG AnxA5-Abs: 20.4%, 95% CI 1.3–50.9%; A5R: 13.2%, 9.7–17.1%), suggesting potential relevance in patients who fulfill clinical APS criteria but test negative for conventional aPL. Among all AnxA5-IgG-positive patients across studies (n = 636), approximately 13% (83/636) were from seronegative APS cohorts, 50.5% (321/636) from seropositive cohorts, and 36.5% (232/636) from mixed/unstratified serologic cohorts. This distribution indicates that while AnxA5 antibodies frequently co-occur with conventional aPL, they also identify a clinically relevant subset of seronegative patients. In the subset of studies that explicitly stratified by conventional aPL status, crude IgG AnxA5-Abs positivity was similar in seropositive and seronegative APS cohorts (approximately one-third of patients in each stratum), whereas cohorts with unstratified serologic status showed lower crude positivity. These descriptive comparisons reflect cohort-level strata rather than individual-level diagnostic performance; however, they reinforce that AnxA5-IgG positivity is not restricted to conventional aPL–positive APS and may be present in clinically defined seronegative disease.

Meta-regression analyses confirmed that obstetric APS phenotype was the strongest independent predictor of AnxA5-Abs positivity (aOR 4.68, 95% CI 1.22–17.91 for Sapporo-defined pregnancy loss and stillbirth), with IgG isotype showing higher prevalence than IgM (aOR 2.08, 95% CI 1.30–3.34). These findings support the hypothesis that AnxA5-related abnormalities reflect clinical phenotype—particularly pregnancy morbidity—rather than conventional serologic profile alone [20,21].

### 4.1 Biological plausibility and mechanistic context

These results reinforce the biological basis of the AnxA5 "anticoagulant shield" hypothesis. AnxA5 normally forms a two-dimensional crystalline lattice over anionic phospholipids on endothelial and trophoblast surfaces, blocking assembly of prothrombinase and tenase complexes and thus limiting thrombin generation [10,22]. High-affinity aPL particularly IgG can disrupt this protective lattice, exposing procoagulant phospholipid surfaces [11,12,22]. This mechanism has been directly demonstrated by atomic force microscopy showing aPL-β_2_GPI immune complexes displacing AnxA5 from phospholipid bilayers [22].

Recent prospective evidence strengthens the clinical relevance of this pathway. Elevated preconception IgG anti-AnxA5 levels have been associated with lower live-birth rates and increased miscarriage risk in women with recurrent pregnancy loss [23], and multiple studies report higher anti-AnxA5 prevalence in obstetric cohorts [24]. Furthermore, anti-AnxA5-Abs can appear independently of conventional aPL, supporting their potential value in seronegative presentations [5].

The A5R functional assay quantifies this disruption by measuring plasma coagulation with and without exogenous AnxA5. Reduced A5R has been linked to adverse clinical outcomes: Wolgast et al. demonstrated significantly lower A5R in patients with thrombosis or pregnancy complications [20], while Pignatelli et al. showed that non-criteria aPL, including those targeting AnxA5, identify high-risk seronegative APS patients [21]. Flow-cytometric competition assays similarly detect antibody-mediated interference with AnxA5–phospholipid binding, with the majority of APS patients testing positive compared with a small proportion of controls [25,26].

Because A5R reflects integrated antibody-mediated procoagulant activity rather than a single epitope, it warrants further evaluation as a composite biomarker with potential advantages over individual antibody measurements. Our findings suggest that A5R positivity identifies a distinct clinical subset predominantly obstetric APS rather than simply tracking conventional serologic status. The IgG-dominant signal for anti-AnxA5 antibodies, combined with functional A5R abnormalities, supports the biological relevance of this pathway in pregnancy morbidity [23,27].

### 4.2 Comparison with prior evidence

Prior systematic reviews of non-criteria antiphospholipid antibodies have reported highly variable prevalence estimates for AnxA5-Abs in APS, with some pooled analyses suggesting positivity in approximately 50% of patients in selected cohorts [28]. However, these estimates were derived from heterogeneous populations and assay platforms, and did not consistently distinguish between clinical phenotypes or serologic subgroups. Our lower overall estimates likely reflect broader inclusion criteria encompassing obstetric-only and seronegative cohorts, stricter positivity definitions, and more heterogeneous assay platforms. By stratifying by clinical phenotype and serologic status, we demonstrate that AnxA5 markers are not ubiquitous across APS but instead show phenotype-specific enrichment, with strongest associations in obstetric disease. Variability in patient selection, assay platforms, and threshold definitions across studies [4,5,12] contributes to the wide ranges observed and underscores the need for standardized methodology. Despite these methodological constraints, the consistent enrichment of IgG AnxA5-Abs antibodies and A5R in Sapporo-defined obstetric APS across multiple analytic approaches supports the biological relevance of AnxA5 pathway disruption in pregnancy morbidity.

### 4.3 Study-level bias and evidence quality

Several sources of bias warrant consideration. Meta-regression identified a significant small-study effect, with smaller cohorts reporting higher odds of AnxA5 positivity (aOR 0.54, 95% CI 0.34–0.86 for studies ≥100 participants). This pattern is consistent with possible publication bias, as smaller studies with positive findings may be more likely to reach publication. However, small-study effects may also reflect clinical enrichment in specialized referral centers, differences in assay implementation, or greater phenotypic homogeneity in narrowly defined cohorts These findings were further supported by sensitivity analyses restricting each study to a single estimate, which yielded consistent results and mitigated concerns regarding within-study dependence.

Formal funnel plot asymmetry testing was not performed because most pooled comparisons included fewer than ten studies and demonstrated substantial heterogeneity (I^2^ frequently >90%), limiting the interpretability of funnel-based methods. The observed small-study effect should therefore be interpreted as suggestive rather than definitive evidence of publication bias. Nonetheless, pooled prevalence estimates may be modestly inflated, and future large, prospective studies using standardized methodologies are needed to provide more stable effect estimates.

Additional sources of potential bias include spectrum bias from tertiary-center recruitment, which may enrich for severe or treatment-refractory phenotypes; residual confounding from autoimmune comorbidities, particularly SLE; and verification bias, as 63.9% of studies had unclear risk of bias for systematically ruling out alternative causes of thrombosis or pregnancy loss. Meta-regression analyses showed that most risk-of-bias domains were not significantly associated with prevalence estimates, suggesting that methodological quality did not strongly drive heterogeneity. However, the persistent high I^2^ values indicate unmeasured sources of heterogeneity or true population variability that limit precision.

### 4.4 Assay heterogeneity and interpretation of pooled estimates

A critical limitation of the current evidence base is substantial heterogeneity in assay platforms, antigen preparations, and positivity thresholds. Studies employed diverse ELISA formats (direct, indirect, sandwich), varying antigen sources (recombinant vs. purified human AnxA5), and non-standardized cut-off values ranging from manufacturer recommendations to study-specific percentile thresholds. Functional A5R assays showed even greater variability, with different coagulation platforms (clot-based vs. chromogenic), AnxA5 concentrations, and resistance ratio calculations.

This methodological diversity directly influences reported prevalence: assay sensitivity and specificity vary substantially between platforms due to conformational epitope differences, and threshold heterogeneity affects classification, with lower cut-offs (e.g., >95th percentile) yielding higher prevalence than higher thresholds (e.g., >99th percentile or >3 SD). The high between-study heterogeneity (I^2^ frequently >90%) and wide prediction intervals likely reflect both biological variability and these methodological differences.

Because insufficient numbers of studies used identical platforms with harmonized thresholds, formal assay-stratified pooling was not feasible. Therefore, pooled prevalence estimates should be interpreted as approximate central tendencies across heterogeneous methodologies rather than definitive diagnostic benchmarks. The wide prediction intervals observed in several analyses suggest that true prevalence in future settings may vary substantially, reinforcing the need for standardized methodology before clinical interpretation. These findings highlight the urgent need for international standardization of AnxA5-related assays—including consensus on platforms, antigen preparations, reference materials, and validated positivity thresholds—before meaningful inter-study comparison or clinical implementation can be considered.

In addition, heterogeneity in antibody titration and quantitative thresholds represents a further important limitation. In contrast to conventional apl, where standardized titers are integral to classification and risk stratification, AnxA5-Abs were reported using widely variable and often non-comparable cutoffs (e.g., fixed unit thresholds, arbitrary units, optical density, fluorescence measures, or study-specific definitions such as mean ± 2 or 3 standard deviations or percentile-based thresholds), and several studies reported only dichotomous positivity. This lack of harmonization precluded assessment of dose–response relationships and may further contribute to between-study heterogeneity.

### 4.5 Additional considerations and broader annexin autoimmunity

Some APS cohorts included patients with SLE or other autoimmune conditions, which may influence autoantibody profiles and contribute to residual confounding. The very high A5R prevalence reported in SLE with APS manifestations (52.0%, 95% CI 0.0–100%) derives from limited data and should be interpreted cautiously. Beyond AnxA5, antibodies targeting other annexin family members—including Annexin II, Annexin XI, and related isoforms—have been described in APS and related autoimmune conditions [29]. Although current data remain limited and heterogeneous, these observations suggest a broader spectrum of annexin-related autoimmunity that may contribute to the procoagulant and inflammatory milieu observed in APS. Further mechanistic and translational studies are warranted to define the specificity and clinical relevance of this annexin autoimmunity profile.

### 4.6 Implications in the Era of 2023 ACR/EULAR classification criteria

The 2023 ACR/EULAR classification criteria for APS represent a shift toward a weighted, risk-stratified framework that prioritizes high-risk aPL profiles and assigns differential weighting to clinical domains [30]. While this approach enhances specificity for research classification, it may result in under-classification of certain APS phenotypes, particularly those with isolated obstetric manifestations or lower-risk serologic profiles. Patients without conventional aPL positivity cannot be classified under the current framework, regardless of clinical presentation.

Our findings are therefore notable in demonstrating enrichment of IgG AnxA5-Abs in Sapporo-defined obstetric APS (21.2% vs 4.0% in controls), including among cohorts with seronegative or non-classical serologic features. This observation raises the possibility that a subset of patients with biologically active pregnancy morbidity potentially mediated through disruption of the AnxA5 anticoagulant shield may not be captured within the 2023 classification framework. This may represent an Annexin A5–mediated obstetric APS phenotype that falls outside current classification criteria. Isotype-specific differences provide additional context for interpreting these findings. In established APS literature, IgG aPL are more consistently associated with thrombotic and obstetric manifestations than IgM isotypes, a distinction reflected in the 2023 ACR/EULAR criteria, where isolated IgM positivity carries limited weight [30]. In our analysis, IgM AnxA5-Abs showed low prevalence and no significant enrichment in obstetric APS, in contrast to IgG antibodies, which demonstrated consistent associations. This pattern raises the possibility that IgG AnxA5-Abs may represent a more clinically relevant signal, whereas IgM findings may reflect lower-affinity or transient immune responses. However, these observations should be interpreted cautiously given the limited reporting of IgM-specific data and variability in assay methodologies.

This potential discordance between classification criteria and underlying pathophysiology warrants careful consideration. Although classification criteria are intended for research standardization rather than clinical diagnosis, biomarker-defined subsets that are biologically active yet classification negative may represent important targets for mechanistic investigation and possibly for future risk stratification. Our findings should therefore be interpreted as hypothesis-generating, suggesting that AnxA5-Abs and A5R may help identify a distinct subset of obstetric APS characterized by AnxA5 pathway disruption.

Further prospective studies are required to determine whether such patients experience comparable pregnancy morbidity and recurrence risk, to evaluate the prognostic value of AnxA5–related biomarkers across isotypes, and to assess whether incorporation of functional or non-criteria biomarkers could refine future APS classification strategies without compromising specificity.

### 4.7 Clinical implications and future directions

Our findings establish biological plausibility and phenotype-specific associations that justify further investigation of AnxA5-related biomarkers, particularly in obstetric APS. However, substantial assay heterogeneity, predominantly retrospective study designs, and evidence of small-study effects limit definitive interpretation. Current evidence remains insufficient to support incorporation of these biomarkers into APS classification criteria or clinical risk stratification frameworks.

We did not formally apply the GRADE framework to rate overall certainty of evidence; however, the predominance of retrospective designs, substantial assay heterogeneity, evidence of small-study effects, and wide confidence intervals would likely result in low-to-moderate certainty ratings for most comparisons.

To advance clinical translation, rigorously designed prospective studies are needed with clearly defined APS and seronegative APS cohorts, harmonized assay protocols, and validated reference materials with predefined positivity thresholds. These studies should evaluate incremental prognostic value beyond established APS markers, demonstrate inter-laboratory reproducibility, and include longitudinal follow-up particularly before, during, and after pregnancy. This will eventually clarify whether temporal changes in AnxA5-related markers predict clinical events or recurrence risk. Ultimately, randomized implementation studies or decision-impact analyses are required to demonstrate that AnxA5-guided management improves clinical outcomes compared with standard care.

International collaborative efforts to develop consensus assay protocols and reference materials, analogous to those undertaken for conventional aPL, are essential prerequisites for clinical implementation.

## 5 Conclusion

IgG anti–AnxA5-Abs and A5R demonstrate phenotype-specific enrichment in APS, with the most consistent associations observed in Sapporo-defined obstetric disease. Their functional basis provides biological plausibility linking antibody-mediated disruption of the annexin anticoagulant shield to pregnancy morbidity. Meta-regression suggests that clinical phenotype particularly obstetric manifestations is a stronger determinant of AnxA5-related abnormalities than conventional serologic profile alone.

However, substantial assay heterogeneity, predominance of retrospective study designs, and evidence of small-study effects limit definitive interpretation. Current evidence remains insufficient to support incorporation of these biomarkers into APS classification or clinical risk stratification frameworks. Standardized assay harmonization and prospective external validation are required before clinical implementation can be considered.

## Funding

HC and ZMT are grateful for institutional salary support from the Biomedical Research Program and the Biostatistics, Epidemiology, and Biomathematics Research Core, both at Weill Cornell Medicine-Qatar.

## CRediT authorship contribution statement

**Laith Alamlih:** Conceptualization, Data curation, Methodology, Project administration, Supervision, Writing – original draft, Writing – review & editing. **Karima Becetti:** Conceptualization, Data curation, Writing – original draft, Writing – review & editing. **Samar Al Emadi:** Conceptualization, Data curation, Supervision, Validation, Writing – original draft, Writing – review & editing. **Eman satti:** Conceptualization, Methodology, Writing – review & editing. **Zahir M. Tag:** Conceptualization, Data curation, Formal analysis, Software, Visualization, Writing – review & editing. **Hiam Chemaitelly:** Conceptualization, Data curation, Formal analysis, Methodology, Software, Writing – review & editing. **Mohammad Abu-Tineh:** Conceptualization, Data curation, Methodology, Writing – original draft, Writing – review & editing. **Mohamad Abufaied:** Conceptualization, Data curation, Methodology, Writing – original draft, Writing – review & editing. **Sa'ad Laws:** Conceptualization, Data curation, Methodology, Writing – review & editing. **Munther Khamashta:** Supervision, Validation, Writing – original draft, Writing – review & editing.

## Declaration of competing interest

The authors declare that they have no known competing financial interests or personal relationships that could have appeared to influence the work reported in this manuscript.

## Data Availability

Data will be made available on request.

## Abbreviations

AnxA5: Annexin A5
AnxA5-Abs: anti–Annexin A5 antibodies
A5R: Annexin A5 resistance
aPL: antiphospholipid antibodies
APS: antiphospholipid syndrome
SNAPS: seronegative antiphospholipid syndrome
SLE: systemic lupus erythematosus
IgG/IgM/IgA: immunoglobulin subclasses
ROB: risk of bias
CI: confidence interval
