# Supplementary material for: Anti-Annexin A5 antibodies and Annexin A5 resistance in antiphospholipid syndrome: A systematic review and meta-analysis
Source: J Transl Autoimmun. 2026 Apr 23;12:100373. doi: 10.1016/j.jtauto.2026.100373 (PMC13158777; doi:10.1016/j.jtauto.2026.100373)
Supplement: Multimedia component 1 [file mmc1.docx]

| **Section and Topic** | **Item #** | **Checklist item** | **Location where item is reported** |
| --- | --- | --- | --- |
| **TITLE** | | |  |
| Title | 1 | Identify the report as a systematic review. | Page 1, Title |
| **ABSTRACT** | | |  |
| Abstract | 2 | See the PRISMA 2020 for Abstracts checklist. | Page 3, Abstract |
| **INTRODUCTION** | | |  |
| Rationale | 3 | Describe the rationale for the review in the context of existing knowledge. | Page 4, Introduction section |
| Objectives | 4 | Provide an explicit statement of the objective(s) or question(s) the review addresses. | Page 4-5, "Objectives of the present study" section |
| **METHODS** | | |  |
| Eligibility criteria | 5 | Specify the inclusion and exclusion criteria for the review and how studies were grouped for the syntheses. | Page 6, "Eligibility Criteria" section (Inclusion/Exclusion) |
| Information sources | 6 | Specify all databases, registers, websites, organisations, reference lists and other sources searched or consulted to identify studies. Specify the date when each source was last searched or consulted. | Page 5-6, "Search Strategy and Study Selection" |
| Search strategy | 7 | Present the full search strategies for all databases, registers and websites, including any filters and limits used. | Page 5-6, "Search Strategy and Study Selection" section; Supplementary Box S1 |
| Selection process | 8 | Specify the methods used to decide whether a study met the inclusion criteria of the review, including how many reviewers screened each record and each report retrieved, whether they worked independently, and if applicable, details of automation tools used in the process. | Page 6, "Search Strategy and Study Selection" section |
| Data collection process | 9 | Specify the methods used to collect data from reports, including how many reviewers collected data from each report, whether they worked independently, any processes for obtaining or confirming data from study investigators, and if applicable, details of automation tools used in the process. | Page 6, "Data Extraction and Management" section |
| Data items | 10a | List and define all outcomes for which data were sought. Specify whether all results that were compatible with each outcome domain in each study were sought (e.g. for all measures, time points, analyses), and if not, the methods used to decide which results to collect. | Page 5, "Study Outcomes and Prioritization" section; Page 6, "Data Extraction and Management" |
|  | 10b | List and define all other variables for which data were sought (e.g. participant and intervention characteristics, funding sources). Describe any assumptions made about any missing or unclear information. | Page 6, "Data Extraction and Management" section; Supplementary Box S3 |
| Study risk of bias assessment | 11 | Specify the methods used to assess risk of bias in the included studies, including details of the tool(s) used, how many reviewers assessed each study and whether they worked independently, and if applicable, details of automation tools used in the process. | Page 6-7, "Risk of Bias and Precision Assessment" section; Supplementary Box S4 |
| Effect measures | 12 | Specify for each outcome the effect measure(s) (e.g. risk ratio, mean difference) used in the synthesis or presentation of results. | Page 7, "Data synthesis and analysis" section |
| Synthesis methods | 13a | Describe the processes used to decide which studies were eligible for each synthesis (e.g. tabulating the study intervention characteristics and comparing against the planned groups for each synthesis (item #5)). | Page 7, "Data synthesis and analysis" section |
|  | 13b | Describe any methods required to prepare the data for presentation or synthesis, such as handling of missing summary statistics, or data conversions. | Page 7, "Data synthesis and analysis" section |
|  | 13c | Describe any methods used to tabulate or visually display results of individual studies and syntheses. | Page 7, "Data synthesis and analysis" section |
|  | 13d | Describe any methods used to synthesize results and provide a rationale for the choice(s). If meta-analysis was performed, describe the model(s), method(s) to identify the presence and extent of statistical heterogeneity, and software package(s) used. | Page 7, "Data synthesis and analysis" section (Freeman-Tukey transformation, Restricted Maximum Likelihood model) |
|  | 13e | Describe any methods used to explore possible causes of heterogeneity among study results (e.g. subgroup analysis, meta-regression). | Page 7, "Data synthesis and analysis" section (meta-regression analyses) |
|  | 13f | Describe any sensitivity analyses conducted to assess robustness of the synthesized results. | Page 7, "Data synthesis and analysis" section; subgroup meta-regression for IgG |
| Reporting bias assessment | 14 | Describe any methods used to assess risk of bias due to missing results in a synthesis (arising from reporting biases). | Not explicitly reported / Not applicable |
| Certainty assessment | 15 | Describe any methods used to assess certainty (or confidence) in the body of evidence for an outcome. | Not explicitly reported / Not applicable |
| **RESULTS** | | |  |
| Study selection | 16a | Describe the results of the search and selection process, from the number of records identified in the search to the number of studies included in the review, ideally using a flow diagram. | Page 7-8, "Search Results and Characteristics of the Studies" section; Figure 1 (PRISMA flow diagram) |
|  | 16b | Cite studies that might appear to meet the inclusion criteria, but which were excluded, and explain why they were excluded. | Figure 1, Page 17; full-text exclusions with reasons |
| Study characteristics | 17 | Cite each included study and present its characteristics. | Page 8, Table S1 |
| Risk of bias in studies | 18 | Present assessments of risk of bias for each included study. | Page 8, "Risk of bias and precision assessments" section; Figure S1 and Figure 2 |
| Results of individual studies | 19 | For all outcomes, present, for each study: (a) summary statistics for each group (where appropriate) and (b) an effect estimate and its precision (e.g. confidence/credible interval), ideally using structured tables or plots. | Table 1, Page 8-9; Table S2; Figure S2 |
| Results of syntheses | 20a | For each synthesis, briefly summarise the characteristics and risk of bias among contributing studies. | Page 8-9, "AnxA5-Abs and A5R: Pooled Mean Prevalence" section; Table 1 |
|  | 20b | Present results of all statistical syntheses conducted. If meta-analysis was done, present for each the summary estimate and its precision (e.g. confidence/credible interval) and measures of statistical heterogeneity. If comparing groups, describe the direction of the effect. | Table 1, Page 8-9; Figure S2 |
|  | 20c | Present results of all investigations of possible causes of heterogeneity among study results. | Page 9-10, "Associations with AnxA5-Abs prevalence" section; Tables 2-4 |
|  | 20d | Present results of all sensitivity analyses conducted to assess the robustness of the synthesized results. | Table 3 (IgG-specific analysis); Page 10 |
| Reporting biases | 21 | Present assessments of risk of bias due to missing results (arising from reporting biases) for each synthesis assessed. | Not explicitly reported / Not applicable |
| Certainty of evidence | 22 | Present assessments of certainty (or confidence) in the body of evidence for each outcome assessed. | Not explicitly reported / Not applicable |
| **DISCUSSION** | | |  |
| Discussion | 23a | Provide a general interpretation of the results in the context of other evidence. | Page 10-12, Discussion section |
|  | 23b | Discuss any limitations of the evidence included in the review. | Page 11-12, Discussion section |
|  | 23c | Discuss any limitations of the review processes used. | Page 11-12, Discussion section |
|  | 23d | Discuss implications of the results for practice, policy, and future research. | Page 12, Conclusion paragraph |
| **OTHER INFORMATION** | | |  |
| Registration and protocol | 24a | Provide registration information for the review, including register name and registration number, or state that the review was not registered. | Page 3, Abstract (Methods); Page 5, Methods (PROSPERO CRD42018099462) |
|  | 24b | Indicate where the review protocol can be accessed, or state that a protocol was not prepared. | Page 5, Methods (PROSPERO CRD42018099462) |
|  | 24c | Describe and explain any amendments to information provided at registration or in the protocol. | Page 5, Methods (updated literature search dates) |
| Support | 25 | Describe sources of financial or non-financial support for the review, and the role of the funders or sponsors in the review. | Page 12, Acknowledgements / Funding section |
| Competing interests | 26 | Declare any competing interests of review authors. | Page 12, "Competing interests" section |
| Availability of data, code and other materials | 27 | Report which of the following are publicly available and where they can be found: template data collection forms; data extracted from included studies; data used for all analyses; analytic code; any other materials used in the review. | Page 12, "Data availability" section |

*From:*  Page MJ, McKenzie JE, Bossuyt PM, Boutron I, Hoffmann TC, Mulrow CD, et al. The PRISMA 2020 statement: an updated guideline for reporting systematic reviews. BMJ 2021;372:n71. doi: 10.1136/bmj.n71. This work is licensed under CC BY 4.0. To view a copy of this license, visit <https://creativecommons.org/licenses/by/4.0/>
