# Supplementary material for: Anti-Annexin A5 antibodies and Annexin A5 resistance in antiphospholipid syndrome: A systematic review and meta-analysis
Source: J Transl Autoimmun. 2026 Apr 23;12:100373. doi: 10.1016/j.jtauto.2026.100373 (PMC13158777; doi:10.1016/j.jtauto.2026.100373)
Supplement: Multimedia component 2 [file mmc2.docx]

**_Supplementary Box S1. Database Search Strategies_**

**PubMed Search Strategy**

(("APS antibodies"[Title/Abstract] OR "ANXA5"[Title/Abstract] OR

"Antiphospholipid Antibodies"[Title/Abstract] OR "annexin"[Title/Abstract] OR

"Annexins"[Title/Abstract] OR "Annexin A5"[Mesh:NoExp] OR "Annexins"[Mesh:NoExp] OR

"Antibodies, Antiphospholipid"[Mesh:NoExp])

AND

("Antiphospholipid Syndrome"[Mesh:NoExp] OR "Thrombosis"[Mesh:NoExp] OR

"Venous Thrombosis"[Mesh:NoExp] OR "Stroke"[Mesh:NoExp] OR

"Myocardial Infarction"[Mesh:NoExp] OR "ST Elevation Myocardial Infarction"[Mesh:NoExp] OR

"Pregnancy Complications"[Mesh:NoExp] OR "Abortion, Spontaneous"[Mesh:NoExp] OR

"Abortion, Habitual"[Mesh:NoExp] OR "Abortion, Missed"[Mesh:NoExp] OR

"Fetal Growth Retardation"[Mesh:NoExp] OR "Pre-Eclampsia"[Mesh:NoExp] OR

"Obstetric Labor, Premature"[Mesh:NoExp] OR "Premature Birth"[Mesh:NoExp] OR

"Pregnancy Complications, Cardiovascular"[Mesh:NoExp] OR

"Pregnancy Complications, Hematologic"[Mesh:NoExp] OR

"Antiphospholipid Syndrome"[Title/Abstract] OR

"Antiphospholipid Antibody Syndrome"[Title/Abstract] OR

"Anti-Phospholipid Antibody Syndrome"[Title/Abstract] OR

"Anti-Phospholipid Syndrome"[Title/Abstract] OR

"Thromboses"[Title/Abstract] OR "Thrombus"[Title/Abstract] OR

"Blood Clot"[Title/Abstract] OR "Strokes"[Title/Abstract] OR

"Myocardial Infarction"[Title/Abstract] OR

"Pregnancy Complications"[Title/Abstract] OR "Miscarriage"[Title/Abstract] OR

"Miscarriages"[Title/Abstract] OR "Fetal Death"[Title/Abstract] OR

"Eclampsia"[Title/Abstract] OR "Preeclampsia"[Title/Abstract] OR

"MI"[Title/Abstract] OR "Pregnancy loss"[Title/Abstract]))

**Embase Search Strategy**

("annexin".ti,ab. OR "Annexins".ti,ab. OR "Placental Anticoagulant Protein I".ti,ab. OR

"ANXA5".ti,ab. OR "Antiphospholipid autoantibodies".ti,ab. OR "APS antibodies".ti,ab.)

AND

(antiphospholipid syndrome/ OR thrombosis/ OR artery thrombosis/ OR catheter thrombosis/ OR

heart atrium thrombosis/ OR microthrombus/ OR thrombus/ OR sagittal sinus thrombosis/ OR

deep vein thrombosis/ OR portal vein thrombosis/ OR brain infarction/ OR heart infarction/ OR

acute heart infarction/ OR non ST segment elevation myocardial infarction/ OR

silent myocardial infarction/ OR ST segment elevation myocardial infarction/ OR

pregnancy complication/ OR maternal hypertension/ OR fetus wastage/ OR missed abortion/ OR

recurrent abortion/ OR spontaneous abortion/ OR intrauterine growth retardation/ OR

small for date infant/ OR preeclampsia/ OR pregnancy disorder/ OR high risk pregnancy/ OR

placenta disorder/ OR "Antiphospholipid Syndrome".ti,ab. OR "Thrombosis".ti,ab. OR

"Thrombus".ti,ab. OR "myocardial infarction".ti,ab. OR "Buerger disease".ti,ab. OR

"Blood Clot".ti,ab. OR "CVA".ti,ab. OR "Early Pregnancy Loss".ti,ab. OR

"preterm delivery".ti,ab. OR "APS".ti,ab. OR "Pregnancy".ti,ab.)

**Supplementary Box S2. APS Clinical Criteria Used for Study Eligibility**

defined by at least one of the following manifestations:

- - **Vascular thrombosis:** Venous and/or arterial thrombosis confirmed by objective imaging or histopathology.
  - **Pregnancy morbidity:**
    - One or more unexplained fetal deaths at ≥10 weeks’ gestation, or
    - One or more premature births of a morphologically normal neonate before 34 weeks’ gestation due to pre-eclampsia, eclampsia, or placental insufficiency, or
    - Three or more consecutive spontaneous pregnancy losses before 10 weeks’ gestation unexplained by other causes.

**Supplementary Box S3. Summary of Extracted Variables**

**Bibliographic information**
• Author(s)
• Year of publication
• Full citation
• Country

**Study characteristics**
• Study design (cross-sectional, prospective, retrospective, case–control)
• Total sample size
• Study setting (hospital-based, community-based, mixed)
• Recruitment method

**Participant demographics**
• Mean or median age
• Sex distribution
• Underlying autoimmune disease status
• Relevant comorbidities

**Clinical variables**
• **Thrombosis:** type (venous, arterial, mixed); number of events; treatment; additional thrombotic risk factors
• **Pregnancy morbidity:** recurrent pregnancy loss, stillbirth, preeclampsia, or other outcomes; number of affected pregnancies; treatment details

**Laboratory variables**
• Conventional APS antibodies: lupus anticoagulant (LA), anticardiolipin (aCL), anti-β₂-glycoprotein I (anti-β₂GPI)
• Non-criteria antibodies: Annexin A5 IgG, Annexin A5 IgM
• Annexin A5 resistance (A5R)

**Outcome variables**
• Number and proportion positive for each Annexin A5 biomarker
• Stratification by clinical phenotype (thrombotic vs obstetric APS)
• Stratification by serologic status (seropositive vs seronegative APS)

**Assay characteristics**
• Type of assay (ELISA, functional resistance assay, flow cytometry)
• Manufacturer
• Cut-off definition
• Reproducibility/validation details

**Supplementary Box S4. Criteria used to determine risk-of-bias classification across the five assessment domains**

The assessment of risk of bias (ROB) was based on five predefined domains. For each domain, studies were graded as having low, high or unclear ROB according to the criteria outlined below.

**APS classification validity:**
Low ROB was assigned when APS was defined using the revised Sapporo/Sydney criteria, with clear documentation of clinical manifestations and, where applicable, laboratory confirmation. High ROB was assigned when non-standard or insufficiently defined criteria were used, or when clinical events were not objectively verified. Unclear ROB was used when APS criteria were mentioned but insufficient detail was available to confirm adherence.

**Manifestation ascertainment:**
Low ROB required objective confirmation of thrombosis (e.g. imaging or histopathology) or pregnancy morbidity (e.g. obstetric documentation or ultrasound). High ROB was assigned when ascertainment relied solely on self-report or unverified clinical judgment. Unclear ROB indicated partial or incomplete description of ascertainment methods.

**Exclusion of alternative causes:**
Low ROB was assigned when studies reported evaluation for alternative explanations (e.g. thrombophilia testing, assessment for non-APS causes of pregnancy loss or thrombosis). High ROB was assigned when no attempt to exclude alternative causes was described. Unclear ROB indicated insufficient detail.

**Assay quality:**
Low ROB required clear reporting of assay methodology, including assay type (ELISA or functional assay), manufacturer or validated in-house protocol, cut-off definition, and information on reproducibility or quality control. High ROB was assigned when assays were inadequately described, unvalidated, or lacked defined cut-offs. Unclear ROB indicated partial reporting without sufficient detail.

**Data completeness:**
Low ROB was defined as ≥80% completeness of outcome data for Annexin A5 biomarkers. High ROB was defined as <80% completeness or a high proportion of missing data without explanation. Unclear ROB was assigned when completeness could not be determined.

**Precision classification:**
Studies with sample sizes ≥100 were considered high precision due to lower random error; those with <100 participants were considered low precision.

**Table.S1. Details of the studies included in the systematic review.**

| Author, year | Study  design | Autoimmune  disease (not excluded) | AnxA5  Method | AnxA5  Biomarker | AnxA5  Cutoff | Unit | APS status | Thrombosis | Obstetric | Thrombosis or  Obstetric | Control |
| --- | --- | --- | --- | --- | --- | --- | --- | --- | --- | --- | --- |
| (1)Mu 2024 | retrospective | Yes | ELISA Ruipin biology | IgG/M | 60 | Ng/ml | Mix | 0 | 217 | 0 | 0 |
| \| (2)Roselli \| 2023 \| \| --- \| --- \| | Cross-sectional | Yes | enzyme-linked immunoassay (Orgentec Diagnostika GmbH, Mainz, Germany) | IgG + M | >10 for IgG and IgM | U/mL | Mix | 300 | 0 | 0 | 0 |
| \| (3)Murad \| 2023 \| \| --- \| --- \| | Cross-sectional | Yes | In House ElIZA assays | Ig G | >1.5 | folds | Mix | 0 | 22 | 0 | 6 |
| \| (4)Liu \| 2022 \| \| --- \| --- \| | retrospective | NA | indirect solid-phase ELISA (HUMAN Diagnostics, Inc, Wiesbaden, GER) | IgG/M/A | 25 | U/ml | Seropositive +seronegative | 0 | 0 | 103 | 172 |
| (5)Anunciación-Llunell 2022 | Cross-sectional | NA | LIA (GA Generic Assays GmbH, Dahlewitz, Germany) | IgG | percentile | percentile | Seropositive | 33 | 41 | 0 | 0 |
| \| (6)Hu \| 2021 \| \| --- \| --- \| | Cross-sectional | Yes | AESKULISA® ELISA Test Kits provided by Aesku. Diagnostics GmbH & Co. KG (Wendelsheim, Germany | IgG + Ig M | 18 | U/m | Seropositive | 0 | 0 | 151 | 90 |
| \| (7)Ho \| 2021 \| \| --- \| --- \| | retrospective | Yes | ELISA Demeditec  AnxA5-Abs  IgG/IgM ELISA (Demeditec Diagnostics, Kiel,  Germany) | IgG+IgM | 8 | U/ml | Mix | 148 | 0 | 0 | 0 |
| \| (8)Khogeer \| 2021 \| \| --- \| --- \| | Prospective | Yes | measured by ELISA (Orgentec Diagnostika, GmbH, Germany). | IgG/M | >20 | NA | Mix | 42 | 24 | 0 | 0 |
| (9)Ilan Volkov 2020 | case–control | Yes | LIA (GA Generic Assays, Germany), | IgG+IgM | positive reactivity for ≥ (+ 1) defined by the 99%. | Percentile | Seropositive | 0 | 0 | 130 | 40 |
| (10) Ferreira, 2020 | Cross-sectional | No | ELISA. AIDA Gmbh, Bad Kreuznach, Germany) | Ig G + Ig M | NA | NA | Seropositive + Seronegative | 0 | 0 | 36 | 20 |
| (11)Gaspersic, 2019 | Prospective | NA | ELISA. (Orgentec Diagnostika GmbH, Mainz, Germany) | IgG + Ig M | NA | NA | Mix | 84 | 0 | 0 | 0 |
| (12) Park, 2019 | Prospective | Yes | Line immunoassay (LIA). (GA Generic Assays GmbH, Dahlewitz, Germany) | IgG + Ig M | NA | NA | Mix | 158 | 0 | 0 | 20 |
| (13) Rigano, 2019 | Cross-sectional | NA | Unknown | IgG/IgM | NA | NA | MIX | 148 | 0 | 0 | 0 |
| (14)Thaler, 2019 | Cross-sectional | Yes | Line immunoassay (LIA)  . (Generic Assays (GA), Dahlewitz, Germany) | IgG + Ig M | NA | NA | Seropositive + Seronegative | 0 | 0 | 53 | 34 |
| (15) Becarevic, 2018 | Cross-sectional | No | ELISA. (IBL International, GMBH Hamburg, Germany) | IgG + Ig M | 12, 18 | U/ml | Seropositive | 0 | 0 | 62 | 0 |
| (16)Akinshina, 2018 | Prospective | Yes | ELISA. (Orgentec Diagnostika Ltd, Germany). | Ig G/M | 6 | U/mL | Mix | 68 | 0 | 0 | 0 |
| (17)Pelusa, 2017 | Retrospective | Yes | ‘in house’ ELISA, utilizing AnxA5  form human placenta as antigen (Sigma, St. Louis, USA) | Ig G | 6 | U/ml | MIX | 24 | 0 | 0 | 0 |
| (18) Carmel-Neiderman, 2017 | Cross-sectional | No | (AESKU Diagnostics GmbH and KG, Wendelsheim, Germany | IgM | Uknown | Uknown | Mix | 209 | 0 | 0 | 54 |
| (19)Zhang, 2017 | Cross-sectional | No | ELISA. (Aesku Diagnostics, Wendelsheim, Germany) | IgG + Ig M | 18, 18 | U/ml | Seropositive + Seronegative | 30 | 30 | 170 | 39 |
| (20)Mekinian, 2016 | Prospective | Yes | ELISA (Theradiag, Croissy Beaubourg, France) | Ig G | 6 | U/ml | seronegative | 52 | 65 | 0 | 31 |
| (21)Avriel, 2016 | Prospective | All SLE | Flow cytometric measurement of AnxA5 binding analyzed by standard two-color FCA using an EPICS XL-MCL instrument (Beckman Coulter, Hialea, FL, USA) | A5R | (1.25) immunofluorescence value that was smaller than the mean of normal controls (+1 SD) | FU | Seropositive | 0 | 0 | 10 | 47 (SLE control) |
| (22)Becarevic, 2016 | Cross-sectional | No | ELISA. (IBL International, GMBH Hamburg, Germany. T) | IgM | 18 | U/ml | seropositive | 45 | 43 | 0 | 0 |
| (23)Khizroeva, 2015 | Cross-sectional | NA | ELISA | Ig G/M | NA | NA | Mix | 0 | 146 | 0 | 0 |
| (24)Maneyro, 2015 | Cross-sectional | NA | ELISA | Ig G/M | NA | NA | Mix | 0 | 30 | 0 | 0 |
| (25)Conti, 2014 | Cross-sectional | yes | ELISA. from INOVA  Diagnostics Inc. (San Diego, CA, USA). | IgG | Uknown | Uknown | Seropositive + Seronegative | 0 | 0 | 49 | 50 (healthy + SLE) |
| (26)Skare, 2014 | Cross-sectional | All SLE | ELISA.(Orgentec®) | IgG | 9 | U/ml | Seropositive | 0 | 0 | 18 | 0 |
| (27)Malickova, 2013 | Cross-sectional | NA | ELISA | Ig G/M/A | NA | NA | seronegative | 0 | 57 | 0 | 0 |
| (28)Pelusa, 2013 | Cross-sectional | yes | EIA | IgG/IgM | Unknown | Unknown | mix |  | 12 |  | 12 |
| (29)Singh, 2013 | Cross-sectional | Yes | ELISA. (Zymutest kit Hyphen Biomed, new Delhi, India) | IgG | aANX IgG antibody values > mean + 2 SD were considered as positive | Unknown | Seropositive | 0 | 0 | 112 | 50 |
| (30)Conti, 2012 | Cross-sectional | yes | ELISA. (in house) | NA | 7.4 | u/ml | negative | 0 | 0 | 36 | 32 |
| (31)Wolgast, 2012 | Cross-sectional | NA | % AnxA5 anticoagulant activity | A5R | lowest quintile of results (<199%) of A5R | % | Seropositive + Seronegative | 151 | 208 | 0 | 140 |
| (32)Akhter, 2012 | Cross-sectional | (all SLE) | AnxA5 anticoagulant ratio | A5R | 3 SD below the mean (140%) of 30 normal healthy control plasmas | % | Mix | 150 | 0 | 0 | 146 (SLE control) |
| (33)Lee, 2011 | Cross-sectional | No | ELISA (ORGENTEC Diagnostika GmbH, Mainz, Germany) | IgG | 8 | U/ml | Mix | 187 | 0 | 0 | 66 |
| (34)Tang, 2011 | Cross-sectional | No | Unknown | IgG/IgM | Unknown | Unknown | Mix | 0 | 51 | 0 |  |
| (35)Hunt, 2011 | Cross-sectional | No | AnxA5 anticoagulant ratio | A5R | minus two standard deviations (SD) of the 30 normal healthy controls | % | Seropositive | 50 | 70 | 0 | 16 |
| (36)Rezk, 2010 | Cross-sectional | No | ELISA. (Orgentec Diagnostika GmbH, Germany). | Ig G +IgM | 7, 15 | U/ml | Mix | 0 | 30 | 0 | 20 |
| (37)GarciaRui, 2010 | Retrospective | NA | NA | NA | Unknown | Unknown | MIX | 0 | 59 | 0 | 68 |
| (38)Wolgast, 2010 | Cross-sectional | NA | Prolongation of coagulation times by AnxA5 | A5R | 210% - the lowest quartile value of A5R - | % | Seropositive + Seronegative | 221 | 0 | 150 | 76 |
| (39) Becarevic, 2008 | Cross-sectional | NO | ELISA. (Orgentec Diagnostika GmbH, Germany). | Ig G + Ig M | 8 | U/ml | seronegative | 40 | 0 | 44 | 0 |
| (40)Tomer, 2007 | Cross-sectional | Yes | Flow cytometric measurement of AnxA5  binding | A5R | Fluorescence value l group )3 standard deviations (SD). | Fluorescence units | Seropositive | 0 | 0 | 66 | 88 |
| (41)Mtiraoui, 2007 | Cross-sectional | NA | ELISA Zymutest kit (Hyphen Biomed, France) | Ig G + Ig M | 13.7 | AU | Mix | 0 | 200 | 0 | 200 |
| (42)Peluso, 2007 | Cross-sectional | yes | (ELISA) immunoenzymatic | IgG | Unknown | Unknown | mix | 0 | 88 | 0 | 0 |
| (43)Rand, 2006 | Cross-sectional | NA | ELISA. (American Diagnostica Inc, Greenwich, CT) | Ig G + Ig M+ A5R | Unknown | AU | Mix | 0 | 70 | 0 | 50 |
| (44)Wu, 2006 | Cross-sectional | NA | ELISA (American Diagnostica Inc, Greenwich, CT) | IgG + Ig M | Unknown | AU | Mix | 0 | 150 | 0 | 90 |
| (45)Zammiti, 2006 | Cross-sectional | NA | Zymutest kit. (Hyphen Biomed) | IgG + Ig M | 20, 20 | U/ml | Mix | 0 | 172 | 0 | 173 |
| (46) Ulcova-Gallova, 2006 | Cross-sectional | NA | ELISA (Sigma, Saint Louis, Missouri, USA) | IgG/IgM | NA | NA | Mix | 0 | 156 | 0 | 0 |
| (47)Bizzaro, 2005 | Cross-sectional | No | Enzyme-linked immunosorbent assays (Orgentec Diagnostika GmbH, Mainz, Germany) | IgG | 5 | U/mL | Seropositive + MIX | 23 | 0 | 25 | 120 |
| (48)Ishikura, 2004 | Cross-sectional | NA | Imuclone ELISA kit (ADI) | Ig G + Ig M | Unknown | Unknown | Mix | 78 | 0 | 0 | 0 |
| (49)Gaspersic, 2003 | Cross-sectional | NO | In house (ELISA) | Ig G/M/A | NA | NA | MIX | 40 | 0 | 0 | 23 |
| (50)Roldan, 2002 | Cross-sectional | No | ELISA. (Diagnostica STAGO, France) | Ig G | NA | NA | MIX | 62 | 0 | 0 | 91(SLE control) |
| (51)Nojima, 2001 | Cross-sectional | (All SLE) | g-Irradiated polystyrene plates (Maxi-Sorp Nunc-Immunoplates; Kamstrup) Coted with AnxA5  (Sigma). | Ig G | 299.8 (exceeded 3 SD of the mean of the 80 controls.) | milliabsorbance units | MIX | 49 | 14 | 0 | 25 |
| (52)Arnold, 2001 | Cross-sectional | NA | IN HOUSE ELISA assay  method by Matsuda et al (1994) | IgG | OD value above the control group third quartile. | OD | Seropositive + Seronegative | 0 | 102 | 0 | 0 |
| (53)Gris, 2000 | Prospective | No | In house (ELISA) | IgG + IgM | 99th percentile of the obtained control values for delta A405=0.25 for IgG  A405=0.295 for IgM | OD | Mix | 0 | 1036 | 0 | 0 |
| (54)Ogawa, 2000 | Cross-sectional | Yes | Immulon 1B (Dynex, Chantilly, VA) (plain plate) + Maxisorp (Nunc, Roskilde, Denmark) (gamma-irradiated plate.) | Ig G + IG M | values greater than the mean + 3 SD for 30 normal control plasma |  | Seropositive | 30 | 0 | 0 | 0 |
| (55)Satoh, 1999 | Cross-sectional | No | (Immulon I, Dynatech, Chantilly, VA, USA) | IgG | Unknown | Unknown | Seropositive | 0 | 0 | 23 | 0 |
| (56)Eschwege, 1998 | Cross-sectional | NA | ELISA | NA | Unknown | Unknown | mix | 0 | 0 | 122 | 100( SLE control) |
| (57)Kaburaki, 1997 | Cross-sectional | (all SLE) | In house (ELISA) | IgG | above 7.4 | u/ml | Mix | 40 | 11 | 0 | 70 (ITP + healthy) |
| (58)Kaburaki, 1995 | Cross-sectional | (all SLE) | Unknown | Ig G | 70U/ml( avarage of level in nomal 45 subject = 6 times standard deviation ) | U/ml | MIX | 40 | 0 | 0 | 0 |
|  | | | | | | | | 2640 | 3065 | 1255 | 2417 |
|  |  |  |  |  |  |  |  | Total: 9377 | | | |

**The subtotal values at the bottom of Table S1 represent the total number of individuals contributing data within each clinical category (thrombosis, pregnancy morbidity, mixed, and control populations). These figures reflect raw patient counts reported in the primary studies and are *not* denominators used for pooled prevalence calculations.**

**Table S2. Characteristics of stratified prevalence measures identified through the systematic review.**

| **Variable** | **Number of prevalence measures** | **%** |
| --- | --- | --- |
| **Study design** |  |  |
| Cross-sectional | 135 | 79.9 |
| Prospective | 18 | 10.7 |
| Retrospective | 12 | 7.1 |
| Case-control | 4 | 2.4 |
| **Population type** |  |  |
| Obstetric | 40 | 23.7 |
| Thrombosis | 45 | 26.6 |
| Mixed^*^ | 30 | 17.8 |
| Control | 54 | 32.0 |
| **Pregnancy type** |  |  |
| Sapporo | 35 | 20.7 |
| Recurrent abortions | 32 | 18.9 |
| Still births | 2 | 1.2 |
| Other/Unspecified | 1 | 0.6 |
| Control | 54 | 32.0 |
| Not applicable | 45 | 26.6 |
| **Thrombosis type** |  |  |
| Arterial | 9 | 5.3 |
| Venous | 12 | 7.1 |
| Mixed^†^ | 53 | 31.4 |
| Control | 54 | 32.0 |
| Not applicable | 41 | 24.3 |
| **Autoimmune disorder** |  |  |
| SLE | 10 | 5.9 |
| Mixed | 74 | 43.8 |
| Control-RA | 2 | 1.2 |
| Control-SLE | 6 | 3.6 |
| Control-mixed | 26 | 15.4 |
| Control-no autoimmune disease | 19 | 11.2 |
| Control all | 1 | 0.6 |
| No autoimmune disease | 31 | 18.3 |
| **APS status** |  |  |
| Seropositive | 40 | 23.7 |
| Seronegative | 17 | 10.1 |
| Mixed | 66 | 39.1 |
| Control-mixed | 45 | 26.6 |
| Control-seropositive | 1 | 0.6 |
| **Biomarker** |  |  |
| IgG | 81 | 47.9 |
| IgM | 44 | 26.0 |
| IgG/IgM/IgA | 18 | 10.7 |
| A5R | 20 | 11.8 |
| Unknown | 6 | 3.6 |
| **Risk of bias assessment** |  |  |
| **Validity of APS criteria** |  |  |
| Low ROB | 108 | 63.9 |
| High ROB | 2 | 1.2 |
| Unclear | 58 | 34.3 |
| Not applicable | 1 | 0.6 |
| **Ascertainment using diagnostics** |  |  |
| Low ROB | 58 | 34.3 |
| High ROB | 6 | 3.6 |
| Unclear | 105 | 62.1 |
| **Ruling out other causes** |  |  |
| Low ROB | 50 | 29.6 |
| High ROB | 11 | 6.5 |
| Unclear | 108 | 63.9 |
| **Consistency in diagnostic methods** |  |  |
| Low ROB | 92 | 54.4 |
| High ROB | 0 | 0.0 |
| Unclear | 77 | 45.6 |
| **Data completeness** |  |  |
| Low ROB | 122 | 72.2 |
| High ROB | 0 | 0.0 |
| Unclear | 47 | 27.8 |
| **Precision (sample size)** |  |  |
| <100 | 125 | 74.0 |
| ≥100 | 44 | 26.0 |

Abbreviations: APS, antiphospholipid syndrome; IgA, immunoglobulin A; IgG, immunoglobulin G; IgM, immunoglobulin M; RA, rheumatoid arthritis; ROB, risk of bias; SLE, systemic lupus erythematosus.

^*^Includes cases originally classified as “obstetric or thrombosis” or “mixed”.

^†^Includes cases originally classified as “arterial or venous” or “mixed”.

**Figure S1. Traffic light plots displaying the risk of bias assessment for studies reporting AnxA5-Abs and A5R prevalence.**

**
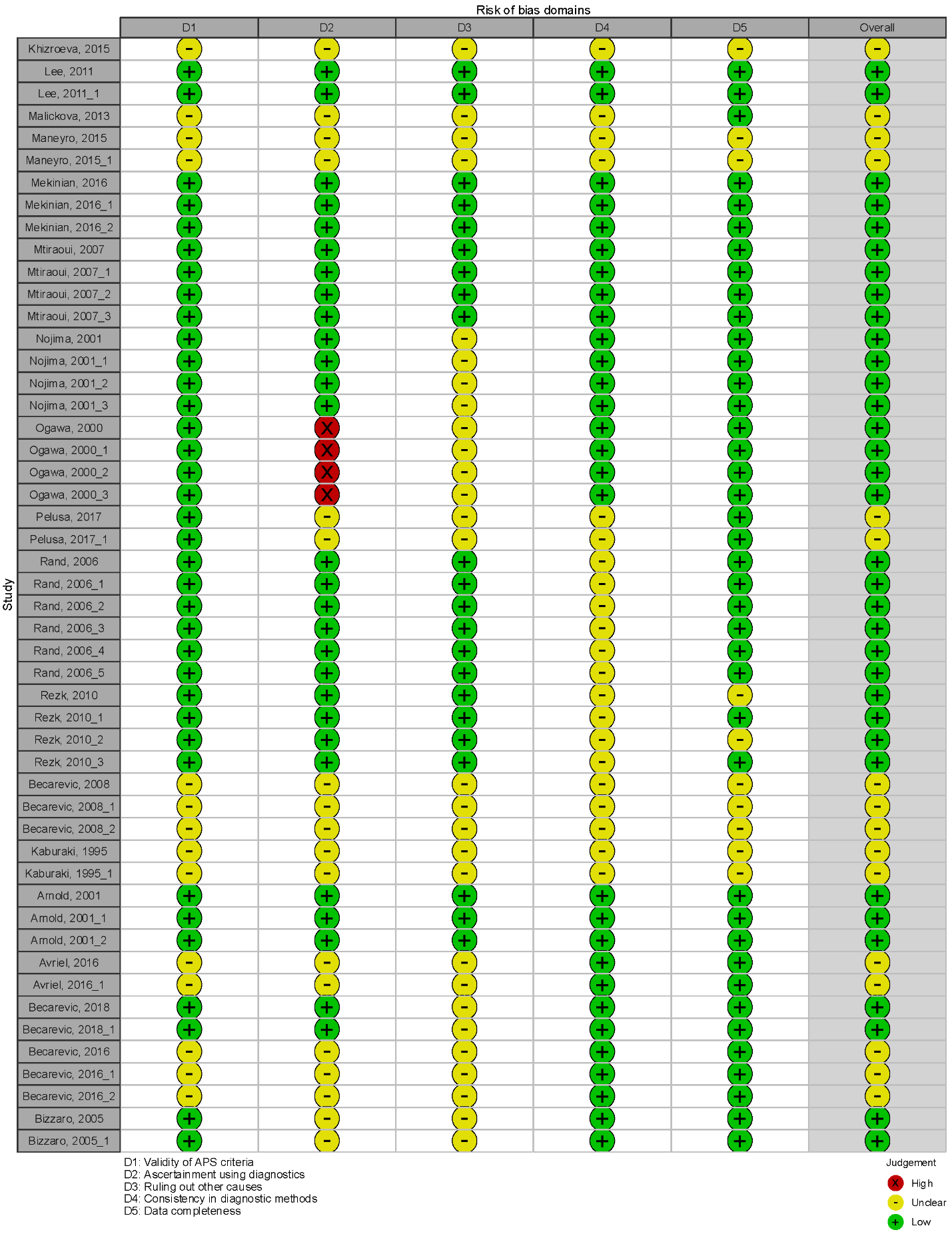
**

**
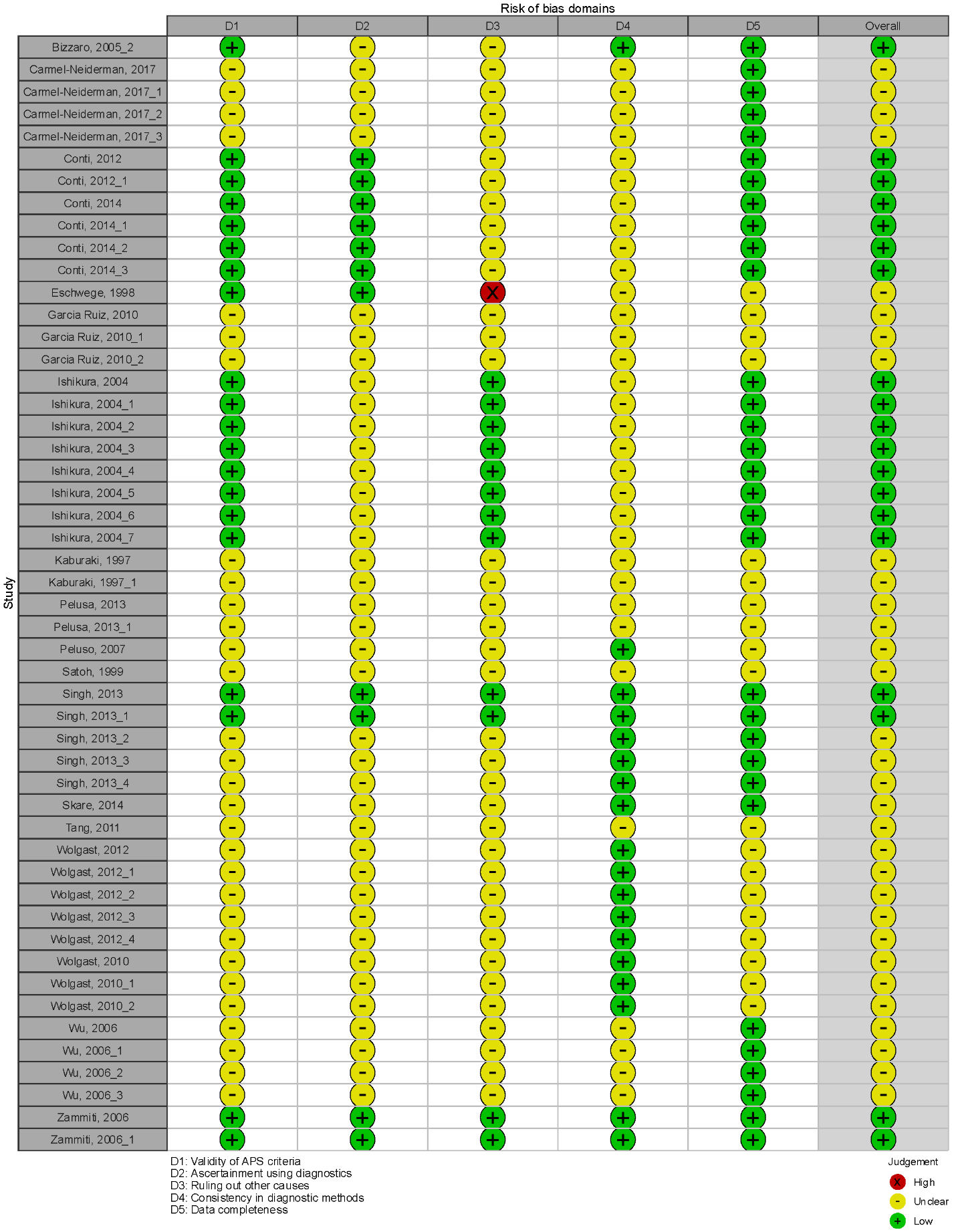
**

**
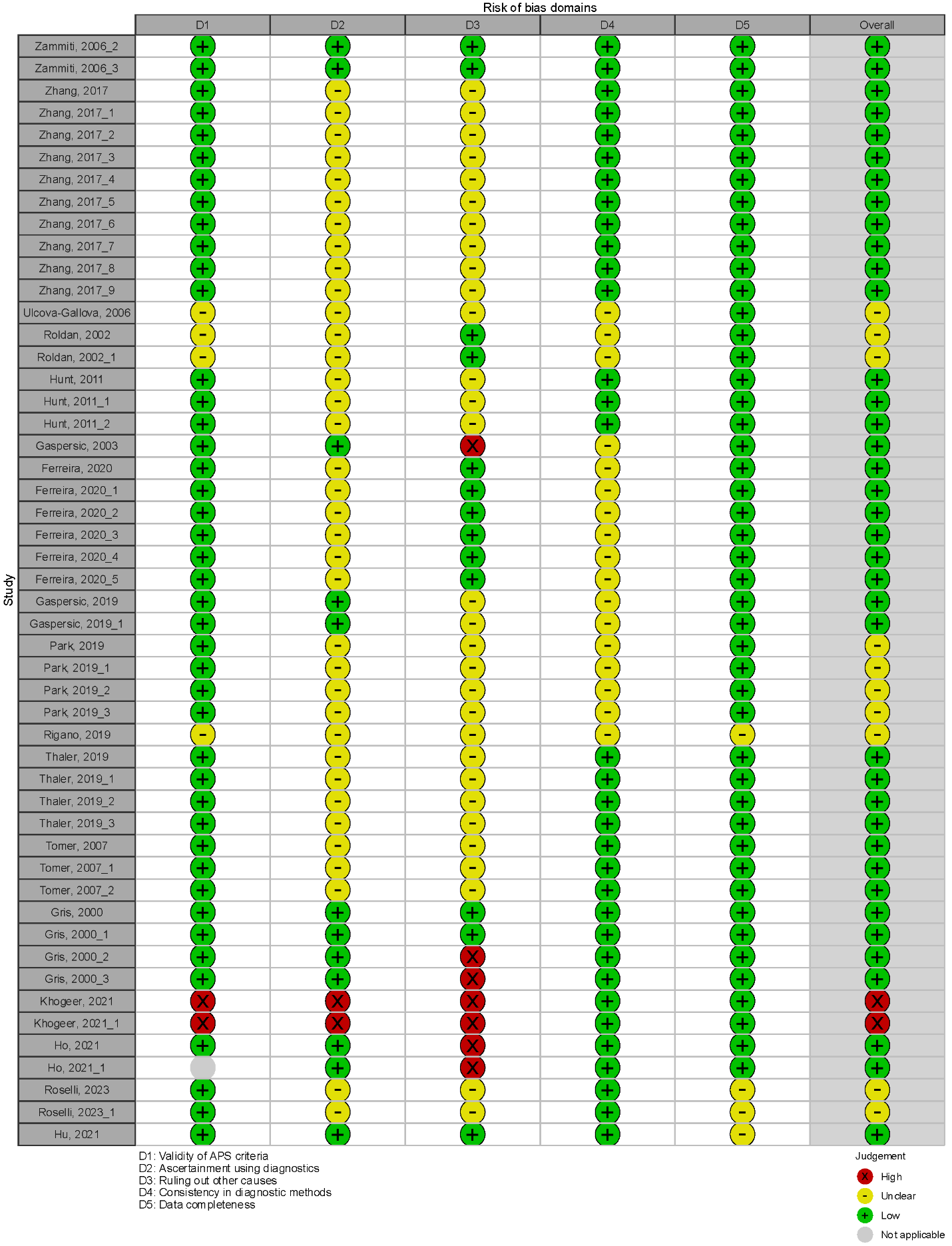
**

**
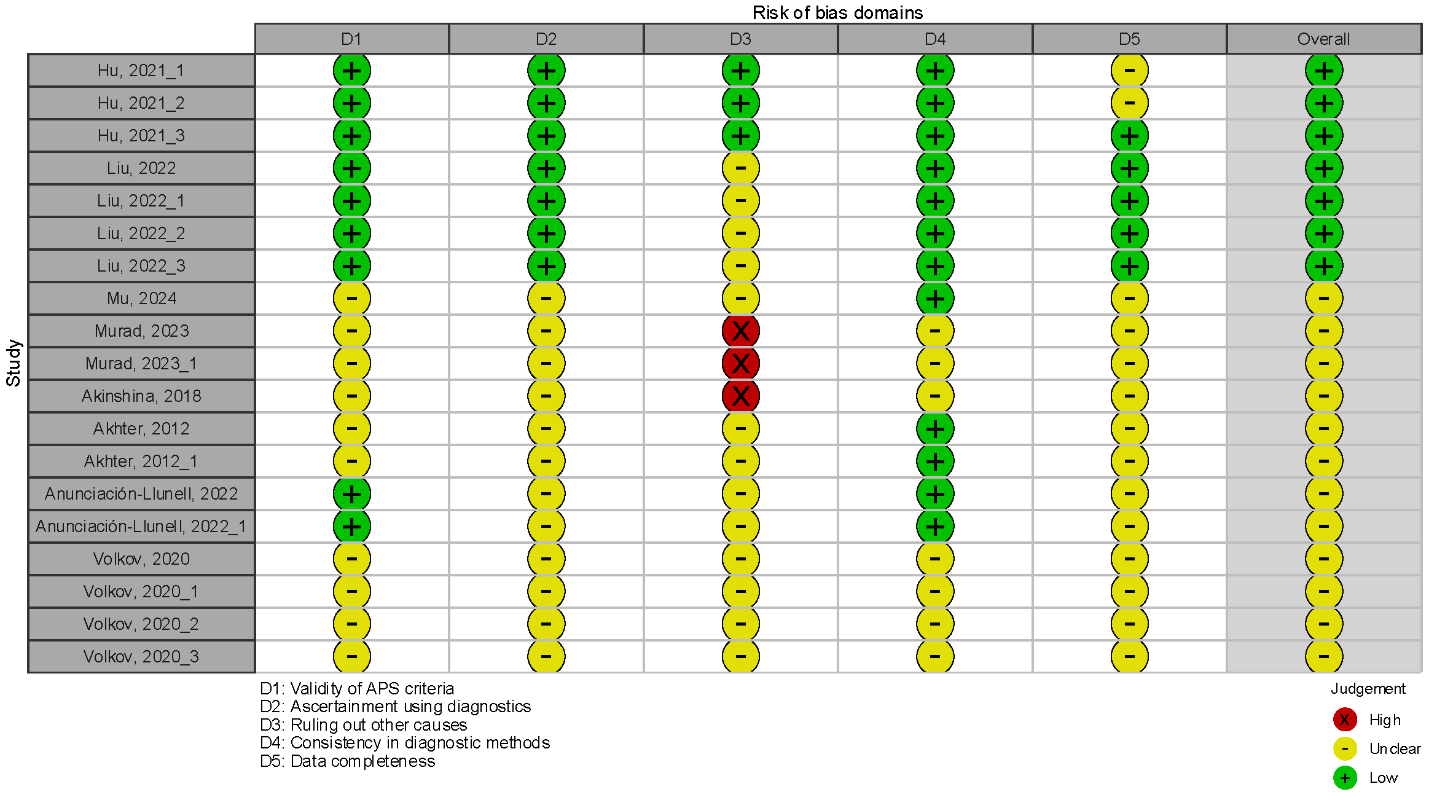
**

Abbreviations: APS, antiphospholipid syndrome.

**Figure S2. Forest plots presenting outcomes of the pooled mean AnxA5-Ab and A5R prevalence.**

1. Population type (IgG obstetric)**.**

**

**

1. Population type (IgM obstetric)

**

**

1. Population type (IgG thrombosis)

**

**

1. Population type (IgM thrombosis)

**

**

1. Population type (IgG control)

**

**

1. Population type (IgM control)

**

**

1. APS status (IgG control)

**

**

1. APS status (IgM control)

**

**

1. Autoimmune disorder (IgG control)

**

**

1. Autoimmune disorder (IgM control)

**

**

1. Autoimmune disorder (IgG control-RA)

**

**

1. Autoimmune disorder (IgG control-SLE)

**

**

1. Autoimmune disorder (IgG control-no autoimmune disease)

**

**

1. Autoimmune disorder (IgM control-no autoimmune disease)

**

**

1. Autoimmune disorder (IgG control-RA and SLE)

**

**

Abbreviations: APS, antiphospholipid syndrome; CI, confidence interval; IgG, immunoglobulin G; IgM, immunoglobulin M; Prev, prevalence; RA, rheumatoid arthritis; SLE, systemic lupus erythematosus; W, weight.

**Table S3. Meta-analyses estimating the pooled mean AnxA5-Ab and A5R prevalence stratified by pregnancy type and thrombosis type.**

| **Populations** | **Studies** | **Sample** | | **Prevalence (%)** | | | | **Heterogeneity measures** | | |
| --- | --- | --- | --- | --- | --- | --- | --- | --- | --- | --- |
|  | Total N | Tested | Positive | Median | Range | Pooled mean | 95% CI | Q^*^ (p-value) | I^2†^ (%, 95% CI) | Prediction interval^‡^ (95% CI) |
| **Pregnancy type** |  |  |  |  |  |  |  |  |  |  |
| Sapporo |  |  |  |  |  |  |  |  |  |  |
| IgG | 20 | 1,031 | 363 | 30.4 | 6.7-61.6 | **31.0** | 17.0-46.9 | 411.3 (p<0.001) | 95.4 (94.0-96.5) | 0.0-96.5 |
| IgM | 10 | 676 | 76 | 8.6 | 4.1-15.8 | **9.9** | 4.2-17.5 | 53.5 (p<0.001) | 83.2 (70.5-90.4) | 0.0-41.6 |
| IgG/IgM/IgA | 2 | 175 | 103 | 55.1 | 49.2-61.1 | **55.8** | 32.6-77.7 | 8.9 (p=0.003) | 88.8 (57.7-97.0) | 0.0-100.0 |
| A5R | 2 | 136 | 91 | 67.7 | 54.6-80.8 | **71.5** | 15.3-100.0 | 50.7 (p<0.001) | 98.0 (95.4-99.2) | 0.0-100.0 |
| Unknown | 1 | 36 | 0 | -- | -- | **0.0^§^** | 0.0-4.7 | -- | -- | -- |
| Recurrent abortions (not Sappor) |  |  |  |  |  |  |  |  |  |  |
| IgG | 11 | 1,810 | 126 | 4.5 | 3.1-24.1 | **13.1** | 2.9-28.6 | 213.9 (p<0.001) | 95.3 (93.2-96.8) | 0.0-80.8 |
| IgM | 8 | 1,641 | 43 | 3.7 | 2.3-7.0 | **3.2** | 1.4-5.5 | 25.1 (p=0.001) | 72.1 (42.8-86.4) | 0.0-11.6 |
| IgG/IgM/IgA | 7 | 663 | 107 | 10.1 | 8.3-19.9 | **13.5** | 6.4-22.5 | 42.6 (p<0.001) | 85.9 (73.0-92.6) | 0.0-46.3 |
| A5R | 5 | 448 | 155 | 48.0 | 27.9-48.6 | **41.3** | 16.5-68.5 | 78.6 (p<0.001) | 94.9 (90.8-97.2) | 0.0-100.0 |
| Unknown | 1 | 25 | 2 | -- | -- | **8.0^§^** | 0.2-22.6 | -- | -- | -- |
| Still births |  |  |  |  |  |  |  |  |  |  |
| IgG | 1 | 11 | 6 | -- | -- | **54.6^§^** | 24.2-83.3 | -- | -- | -- |
| IgM | -- | -- | -- | -- | -- | **--** | -- | -- | -- | -- |
| IgG/IgM/IgA | -- | -- | -- | -- | -- | **--** | -- | -- | -- | -- |
| A5R | r-- | -- | -- | -- | -- | **--** | -- | -- | -- | -- |
| Unknown | 1 | 34 | 3 | -- | -- | **8.8^§^** | 1.2-21.2 | -- | -- | -- |
| Other/Unspecified |  |  |  |  |  |  |  |  |  |  |
| IgG | -- | -- | -- | -- | -- | **--** | -- | -- | -- | -- |
| IgM | -- | -- | -- | -- | -- | **--** | -- | -- | -- | -- |
| IgG/IgM/IgA | 1 | 30 | 6 | -- | -- | **20.0^§^** | 7.3-36.5 | -- | -- | -- |
| A5R | -- | -- | -- | -- | -- | **--** | -- | -- | -- | -- |
| Unknown | -- | -- | -- | -- | -- | **--** | -- | -- | -- | -- |
| Control |  |  |  |  |  |  |  |  |  |  |
| IgG | 27 | 1,542 | 77 | 4.2 | 0.0-12.5 | **4.0** | 1.7-7.1 | 125.0 (p<0.001) | 79.2 (70.3-85.4) | 0.0-23.3 |
| IgM | 14 | 960 | 35 | 4.0 | 0.0-7.9 | **3.1** | 0.9-6.2 | 46.4 (p<0.001) | 72.0 (52.1-83.7) | 0.0-17.2 |
| IgG/IgM/IgA | 3 | 184 | 77 | 16.0 | 12.2-35.9 | **26.6** | 4.1-58.1 | 32.1 (p<0.001) | 93.8 (85.2-97.4) | 0.0-100.0 |
| A5R | 8 | 563 | 84 | 12.2 | 7.9-33.3 | **17.5** | 7.8-29.7 | 54.4 (p<0.001) | 87.1 (76.8-92.9) | 0.0-63.8 |
| Unknown | 2 | 100 | 3 | 2.2 | 1.1-3.3 | **1.9** | 0.0-8.1 | 1.7 (p=0.191) | 41.4 (--) | 0.0-96.3 |
| **Thrombosis type** |  |  |  |  |  |  |  |  |  |  |
| Arterial |  |  |  |  |  |  |  |  |  |  |
| IgG | 6 | 594 | 42 | 2.3 | 1.3-11.2 | **5.8** | 0.4-15.5 | 49.8 (p<0.001) | 90.0 (80.9-94.7) | 0.0-48.1 |
| IgM | 3 | 320 | 1 | 0.0 | 0.0-1.9 | **0.0** | 0.0-1.1 | 3.7 (p=0.155) | 46.4 (0.0-84.2) | 0.0-7.2 |
| IgG/IgM/IgA | -- | -- | -- | -- | -- | **--** | -- | -- | -- | -- |
| A5R | -- | -- | -- | -- | -- | **--** | -- | -- | -- | -- |
| Unknown | -- | -- | -- | -- | -- | **--** | -- | -- | -- | -- |
| Arterial-MI |  |  |  |  |  |  |  |  |  |  |
| IgG | 1 | 22 | 0 | -- | -- | **0.0^§^** | 0.0-7.8 | -- | -- | -- |
| IgM | -- | -- | -- | -- | -- | **--** | -- | -- | -- | -- |
| IgG/IgM/IgA | -- | -- | -- | -- | -- | **--** | -- | -- | -- | -- |
| A5R | -- | -- | -- | -- | -- | **--** | -- | -- | -- | -- |
| Unknown | -- | -- | -- | -- | -- | **--** | -- | -- | -- | -- |
| Arterial-stroke |  |  |  |  |  |  |  |  |  |  |
| IgG | 3 | 480 | 30 | 1.4 | 1.3-7.7 | **4.3** | 0.0-13.9 | 29.8 (p<0.001) | 93.3 (83.8-97.2) | 0.0-64.6 |
| IgM | 2 | 293 | 0 | 0.0 | 0.0-0.0 | **0.0** | 0.0-0.6 | 0.1 (p=0.759) | 0.0 (--) | 0.0-15.7 |
| IgG/IgM/IgA | -- | -- | -- | -- | -- | **--** | -- | -- | -- | -- |
| A5R | -- | -- | -- | -- | -- | **--** | -- | -- | -- | -- |
| Unknown | -- | -- | -- | -- | -- | **--** | -- | -- | -- | -- |
| Venous |  |  |  |  |  |  |  |  |  |  |
| IgG | 5 | 263 | 34 | 20.8 | 11.1-26.3 | **16.1** | 7.4-27.0 | 16.7 (p=0.002) | 76.0 (41.4-90.2) | 0.0-51.7 |
| IgM | 4 | 244 | 7 | 3.8 | 1.9-6.7 | **2.9** | 0.1-7.8 | 5.5 (p=0.140) | 45.2 (0.0-81.7) | 0.0-20.2 |
| IgG/IgM/IgA | 2 | 216 | 23 | 12.5 | 10.0-15.1 | **11.6** | 3.5-23.5 | 4.7 (p=0.030) | 78.7 (7.4-95.1) | 0.0-100.0 |
| A5R | -- | -- | -- | -- | -- | **--** | -- | -- | -- | -- |
| Unknown | 1 | 122 | 1 | -- | -- | **0.8^§^** | 0.0-3.5 | -- | -- | -- |
| Mixed^**^ |  |  |  |  |  |  |  |  |  |  |
| IgG | 37 | 2,406 | 393 | 20.8 | 3.3-30.4 | **17.1** | 11.0-24.1 | 547.3 (p<0.001) | 93.4 (91.8-94.7) | 0.0-66.2 |
| IgM | 21 | 1,746 | 113 | 5.6 | 1.5-13.6 | **7.0** | 2.8-12.7 | 189.8 (p<0.001) | 89.5 (85.3-92.4) | 0.0-42.5 |
| IgG/IgM/IgA | 7 | 560 | 143 | 12.6 | 7.5-30.5 | **21.0** | 7.3-39.0 | 157.7 (p<0.001) | 96.2 (94.1-97.6) | 0.0-84.1 |
| A5R | 7 | 832 | 234 | 48.0 | 16.5-53.0 | **38.4** | 19.7-59.0 | 125.2 (p<0.001) | 95.2 (92.3-97.0) | 0.0-96.8 |
| Unknown | 2 | 158 | 1 | 0.4 | 0.2-0.6 | **0.4** | 0.0-2.5 | 0.1 (p=0.779) | 0.0 (--) | 0.0-32.2 |
| Control |  |  |  |  |  |  |  |  |  |  |
| IgG | 27 | 1,542 | 77 | 4.2 | 0.0-12.5 | **4.0** | 1.7-7.1 | 125.0 (p<0.001) | 79.2 (70.3-85.4) | 0.0-23.3 |
| IgM | 14 | 960 | 35 | 4.0 | 0.0-7.9 | **3.1** | 0.9-6.2 | 46.4 (p<0.001) | 72.0 (52.1-83.7) | 0.0-17.2 |
| IgG/IgM/IgA | 3 | 184 | 77 | 16.0 | 12.2-35.9 | **26.6** | 4.1-58.1 | 32.1 (p<0.001) | 93.8 (85.2-97.4) | 0.0-100.0 |
| A5R | 8 | 563 | 84 | 12.2 | 7.9-33.3 | **17.5** | 7.8-29.7 | 54.4 (p<0.001) | 87.1 (76.8-92.9) | 0.0-63.8 |
| Unknown | 2 | 100 | 3 | 2.2 | 1.1-3.3 | **1.9** | 0.0-8.1 | 1.7 (p=0.191) | 41.4 (--) | 0.0-96.3 |

Abbreviations: APS, antiphospholipid syndrome; CI, confidence interval; IgA, immunoglobulin A; IgG, immunoglobulin G; IgM, immunoglobulin M; MI, myocardial infarction;

RA, rheumatoid arthritis; ROB, risk of bias; SLE, systemic lupus erythematosus.

A minimum of two population groups was required to conduct a meta-analysis.

^*^Q: Cochran’s Q statistic, a measure used to assess the presence of heterogeneity in effect sizes (here, prevalence) across studies.

^†^I^2^: A measure indicating the proportion of total variation across studies attributable to heterogeneity in effect sizes (here, prevalence) rather than chance.

^‡^Prediction interval: The estimated 95% interval representing the distribution of true effect sizes (here, prevalence) around the estimated mean.

^§^Point estimate derived from a single study.

^**^Includes all arterial, venous, and mixed thrombosis cases.

**Table S4. Results of sensitivity meta-regression analyses for the association with AnxA5-Abs prevalence, including only single studies and studies with case–control data.**

| **Characteristics** | **Measures** | **Samples** | **Univariable analysis** | | | **Multivariable analysis** | | | |
| --- | --- | --- | --- | --- | --- | --- | --- | --- | --- |
|  |  |  |  |  |  | **Model 1^*^** | | **Model 2^*^** | |
|  | N | Tested | OR (95% CI) | p-value | Global p-value^†^ | aOR (95% CI) | p-value^‡^ | aOR (95% CI) | p-value^‡^ |
| Population type |  |  |  |  |  |  |  |  |  |
| Control | 24 | 1,475 | 1.00 |  | 0.001 | 1.00 |  | 1.00 |  |
| Sapporo or still birth | 16 | 767 | 5.92 (2.16-16.19) | 0.001 |  | 1.18 (0.20-7.07) | 0.853 | 2.48 (0.38-16.20) | 0.342 |
| Other pregnancies (not Sapporo) | 18 | 1,974 | 1.68 (0.67-4.23) | 0.273 |  | 0.45 (0.09-2.16) | 0.319 | 1.47 (0.21-10.39) | 0.699 |
| Thrombosis | 17 | 1,707 | 0.96 (0.37-2.49) | 0.936 |  | 0.31 (0.06-1.49) | 0.144 | 0.91 (0.14-6.04) | 0.921 |
| Biomarker |  |  |  |  |  |  |  |  |  |
| IgM | 1 | 43 | 1.00 |  | 0.239 | -- | -- | -- | -- |
| IgG | 55 | 4,445 | 1.54 (0.07-33.20) | 0.783 |  | -- | -- | -- | -- |
| IgG/IgM/IgA | 14 | 1,143 | 2.48 (0.11-57.37) | 0.572 |  | -- | -- | -- | -- |
| Unknown | 5 | 292 | 0.36 (0.01-11.71) | 0.565 |  | -- | -- | -- | -- |
| Autoimmune disorder |  |  |  |  |  |  |  |  |  |
| Control-mixed^§^ | 21 | 1,266 | 1.00 |  | 0.031 | -- | -- | 1.00 |  |
| Control-SLE | 3 | 209 | 4.00 (0.65-24.60) | 0.135 |  | -- | -- | 5.65 (1.19-26.78) | 0.029 |
| Mixed^§^ | 46 | 4,342 | 2.27 (0.91-5.69) | 0.080 |  | -- | -- | 0.28 (0.07-1.06) | 0.060 |
| SLE | 5 | 106 | 9.45 (2.00-44.64) | 0.005 |  | -- | -- | Omitted |  |
| Autoimmune disorder |  |  |  |  |  |  |  |  |  |
| Control-mixed^¶^ | 17 | 1,168 | 1.00 |  | 0.057 | -- | -- | -- | -- |
| Control-no autoimmune disease | 7 | 307 | 0.17 (0.02-1.19) | 0.074 |  | -- | -- | -- | -- |
| Mixed^¶^ | 39 | 3,186 | 1.76 (0.69-4.46) | 0.235 |  | -- | -- | -- | -- |
| No autoimmune disease | 12 | 1,262 | 0.88 (0.26-3.00) | 0.833 |  | -- | -- | -- | -- |
| APS status |  |  |  |  |  |  |  |  |  |
| Control | 20 | 1,207 | 1.00 |  | 0.005 | 1.00 |  | 1.00 |  |
| Seronegative | 9 | 357 | 9.35 (2.52-34.72) | 0.001 |  | 11.66 (1.52-89.66) | 0.018 | 28.04 (3.71-211.90) | 0.001 |
| Seropositive | 9 | 545 | 4.27 (1.23-14.75) | 0.022 |  | 4.35 (0.58-32.79) | 0.153 | 8.96 (1.24-64.85) | 0.030 |
| Mixed | 37 | 3,814 | 2.04 (0.84-4.93) | 0.115 |  | 4.21 (0.84-21.16) | 0.081 | 6.67 (1.35-32.92) | 0.020 |
| Year of data collection (median) | 75 | 5,923 | 1.01 (0.96-1.06) | 0.738 | 0.738 |  |  |  |  |
| Validity of APS criteria |  |  |  |  |  |  |  |  |  |
| Low ROB | 42 | 3,601 | 1.00 |  | 0.643 | -- | -- | -- | -- |
| High ROB | 1 | 24 | 0.90 (0.03-23.68) | 0.951 |  | -- | -- | -- | -- |
| Unclear | 31 | 2,150 | 1.45 (0.66-3.18) | 0.355 |  | -- | -- | -- | -- |
| Ascertainment using diagnostics |  |  |  |  |  |  |  |  |  |
| Low ROB | 30 | 2,949 | 1.00 |  | 0.680 | -- | -- | -- | -- |
| High ROB | 2 | 48 | 2.49 (0.26-24.01) | 0.430 |  | -- | -- | -- | -- |
| Unclear | 43 | 2,926 | 1.25 (0.56-2.77) | 0.585 |  | -- | -- | -- | -- |
| Ruling out other causes |  |  |  |  |  |  |  |  |  |
| Low ROB | 23 | 2,294 | 1.00 |  | 0.701 | -- | -- | -- | -- |
| High ROB | 7 | 440 | 1.80 (0.45-7.13) | 0.403 |  | -- | -- | -- | -- |
| Unclear | 45 | 3,189 | 1.22 (0.51-2.91) | 0.654 |  | -- | -- | -- | -- |
| Consistency in diagnostic methods |  |  |  |  |  |  |  |  |  |
| Low ROB | 32 | 3,328 | 1.00 |  | 0.525 | -- | -- | -- | -- |
| Unclear | 43 | 2,595 | 0.78 (0.36-1.68) | 0.525 |  | -- | -- | -- | -- |
| Data completeness |  |  |  |  |  |  |  |  |  |
| Low ROB | 51 | 4,031 | 1.00 |  | 0.143 | 1.00 |  | 1.00 |  |
| Unclear | 24 | 1,892 | 1.80 (0.82-3.96) | 0.143 |  | 1.73 (0.84-3.57) | 0.138 | 1.69 (0.84-3.37) | 0.138 |
| Sample size |  |  |  |  |  |  |  |  |  |
| <100 | 55 | 2,216 | 1.00 |  | 0.145 | 1.00 |  | 1.00 |  |
| ≥100 | 20 | 3,707 | 0.55 (0.25-1.22) | 0.145 |  | 0.67 (0.33-1.38) | 0.282 | 0.77 (0.38-1.56) | 0.461 |

Abbreviations: aOR, adjusted odds ratio; APS, antiphospholipid syndrome; CI, confidence interval; IgA, immunoglobulin A; IgG, immunoglobulin G; IgM, immunoglobulin M; OR, odds ratio; RA, rheumatoid arthritis; ROB, risk of bias; SLE, systemic lupus erythematosus.

^*^Two multivariable logistic regression models were constructed. The first model excluded autoimmune disorder due to collinearity, whereas the second model retained this variable.

^†^Covariates with p-value ≤0.2 in the univariable analysis were included in the multivariable analysis.

^‡^Covariates with p-value <0.05 in the multivariable analysis were considered as showing statistically significant evidence for an association with AnxA5-Abs prevalence.

^§^Mixed population includes controls and cases with no autoimmune disease, RA, and mixed status.

^¶^Mixed population includes controls and cases with SLE, RA, and mixed status.

**Table S5. Results of sensitivity meta-regression analyses for the association with AnxA5-Abs prevalence as assessed using IgG, including only single studies and studies with case–control data.**

| **Characteristics** | **Measures** | **Samples** | **Univariable analysis** | | | **Multivariable analysis** | |
| --- | --- | --- | --- | --- | --- | --- | --- |
|  |  |  |  |  |  |  |  |
|  | N | Tested | OR (95% CI) | p-value | Global p-value^*^ | aOR (95% CI) | p-value^†^ |
| Population type |  |  |  |  |  |  |  |
| Control | 20 | 1,241 | 1.00 |  | 0.002 | 1.00 |  |
| Sapporo or still birth | 13 | 637 | 8.36 (2.56-27.30) | <0.001 |  | 10.82 (1.04-112.24) | 0.046 |
| Other pregnancies (not Sapporo) | 9 | 1,238 | 1.83 (0.53-6.37) | 0.342 |  | 3.21 (0.37-28.22) | 0.293 |
| Thrombosis | 13 | 1,329 | 1.19 (0.38-3.74) | 0.763 |  | 2.73 (0.32-23.58) | 0.362 |
| Autoimmune disorder |  |  |  |  |  |  |  |
| Control-mixed^‡^ | 17 | 1,032 | 1.00 |  | 0.209 | -- | -- |
| Control-SLE | 3 | 209 | 5.69 (0.77-42.18) | 0.089 |  | -- | -- |
| Mixed^‡^ | 30 | 3,098 | 3.19 (1.02-9.94) | 0.046 |  | -- | -- |
| SLE | 5 | 106 | 13.27 (2.38-74.08) | 0.003 |  | -- | -- |
| Autoimmune disorder |  |  |  |  |  |  |  |
| Control-mixed^§^ | 13 | 934 | 1.00 |  | 0.086 | -- | -- |
| Control-no autoimmune disease | 7 | 307 | 0.18 (0.02-1.56) | 0.119 |  | -- | -- |
| Mixed^§^ | 26 | 2,076 | 2.17 (0.67-7.07) | 0.197 |  | -- | -- |
| No autoimmune disease | 9 | 1,128 | 1.19 (0.25-5.63) | 0.824 |  | -- | -- |
| APS status |  |  |  |  |  |  |  |
| Control | 17 | 1,095 | 1.00 |  | 0.022 | 1.00 |  |
| Seronegative | 6 | 204 | 10.74 (1.93-59.87) | 0.007 |  | 1.27 (0.09-18.44) | 0.859 |
| Seropositive | 8 | 502 | 4.92 (1.14-21.13) | 0.032 |  | 0.34 (0.02-4.95) | 0.427 |
| Mixed | 24 | 2,644 | 1.88 (0.63-5.57) | 0.257 |  | 0.50 (0.06-4.34) | 0.527 |
| Year of data collection (median) | 55 | 4,445 | 0.98 (0.93-1.04) | 0.544 | 0.544 | -- | -- |
| Validity of APS criteria |  |  |  |  |  |  |  |
| Low ROB | 36 | 3,189 | 1.00 |  | 0.290 | -- | -- |
| Unclear | 18 | 1,108 | 1.76 (0.62-5.04) | 0.290 |  | -- | -- |
| Ascertainment using diagnostics |  |  |  |  |  |  |  |
| Low ROB | 24 | 2,537 | 1.00 |  | 0.488 | -- | -- |
| High ROB | 1 | 24 | 6.71 (0.24-190.46) | 0.265 |  | -- | -- |
| Unclear | 30 | 1,884 | 1.37 (0.50-3.74) | 0.539 |  | -- | -- |
| Ruling out other causes |  |  |  |  |  |  |  |
| Low ROB | 23 | 2,294 | 1.00 |  | 0.207 | -- | -- |
| High ROB | 3 | 186 | 6.19 (0.79-48.33) | 0.082 |  | -- | -- |
| Unclear | 29 | 1,965 | 1.08 (0.39-3.00) | 0.881 |  | -- | -- |
| Consistency in diagnostic methods |  |  |  |  |  |  |  |
| Low ROB | 27 | 2,862 | 1.00 |  | 0.950 | -- | -- |
| Unclear | 28 | 1,583 | 0.97 (0.36-2.60) | 0.950 |  | -- | -- |
| Data completeness |  |  |  |  |  |  |  |
| Low ROB | 42 | 3,461 | 1.00 |  | 0.016 | 1.00 |  |
| Unclear | 13 | 984 | 3.69 (1.28-10.69) | 0.016 |  | 3.85 (1.40-10.63) | 0.009 |
| Sample size |  |  |  |  |  |  |  |
| <100 | 41 | 1,649 | 1.00 |  | 0.057 | 1.00 |  |
| ≥100 | 14 | 2,796 | 0.37 (0.14-1.03) | 0.057 |  | 0.38 (0.16-0.92) | 0.033 |

Abbreviations: aOR, adjusted odds ratio; APS, antiphospholipid syndrome; CI, confidence interval; IgG, immunoglobulin G; RA, rheumatoid arthritis; ROB, risk of bias; SLE, systemic lupus erythematosus.

^*^Covariates with p-value ≤0.2 in the univariable analysis were included in the multivariable analysis.

^†^Covariates with p-value <0.05 in the multivariable analysis were considered as showing statistically significant evidence for an association with AnxA5-Abs prevalence.

^‡^Mixed population includes controls and cases with RA, mixed status, and no autoimmune disease.

^§^Mixed population includes controls and cases with RA, SLE, and mixed status.

**Table S6. Results of sensitivity meta-regression analyses for the association with A5R prevalence, including only single studies and studies with case–control data.**

| **Characteristics** | **Measures** | **Samples** | **Univariable analysis** | | | **Multivariable analysis** | |
| --- | --- | --- | --- | --- | --- | --- | --- |
|  |  |  |  |  |  |  |  |
|  | N | Tested | OR (95% CI) | p-value | Global p-value^*^ | aOR (95% CI) | p-value^†^ |
| Population type |  |  |  |  |  |  |  |
| Control | 7 | 514 | 1.00 |  | 0.099 | 1.00 |  |
| Sapporo or still birth | 2 | 136 | 12.89 (1.51-110.30) | 0.020 |  | 0.55 (0.00-505.64) | 0.865 |
| Other pregnancies (not Sapporo) | 4 | 337 | 3.16 (0.57-17.33) | 0.186 |  | 1.13 (0.01-107.86) | 0.958 |
| Thrombosis | 1 | 150 | 0.93 (0.06-15.12) | 0.960 |  | 1.56 (0.04-58.43) | 0.810 |
| Autoimmune disorder |  |  |  |  |  |  |  |
| Control-mixed^‡^ | 4 | 282 | 1.00 |  | 0.290 | -- | -- |
| Control-SLE | 3 | 232 | 2.66 (0.29-24.58) | 0.389 |  | -- | -- |
| Mixed^‡^ | 5 | 463 | 6.12 (0.86-43.51) | 0.070 |  | -- | -- |
| SLE | 2 | 160 | 6.31 (0.44-90.10) | 0.175 |  | -- | -- |
| Autoimmune disorder |  |  |  |  |  |  |  |
| Control-mixed^§^ | 6 | 498 | 1.00 |  | 0.339 | -- | -- |
| Control-no autoimmune disease | 1 | 16 | 2.94 (0.11-79.14) | 0.520 |  | -- | -- |
| Mixed^§^ | 6 | 553 | 4.92 (0.87-27.81) | 0.072 |  | -- | -- |
| No autoimmune disease | 1 | 70 | 3.47 (0.15-82.45) | 0.442 |  | -- | -- |
| APS status |  |  |  |  |  |  |  |
| Control | 6 | 368 | 1.00 |  | 0.014 | 1.00 |  |
| Seronegative | 1 | 107 | 0.36 (0.03-4.62) | 0.429 |  | 1.08 (0.00-780.48) | 0.982 |
| Seropositive | 4 | 296 | 9.79 (2.01-47.73) | 0.005 |  | 12.25 (0.03-4908.15) | 0.412 |
| Mixed | 3 | 366 | 1.19 (0.23-6.25) | 0.836 |  | 1.87 (0.06-58.45) | 0.721 |
| Year of data collection (median) | 14 | 1,137 | 0.94 (0.72-1.23) | 0.646 | 0.646 | -- | -- |
| Validity of APS criteria |  |  |  |  |  |  |  |
| Low ROB | 6 | 311 | 1.00 |  | 0.086 | 1.00 |  |
| Unclear | 8 | 826 | 0.28 (0.06-1.20) | 0.086 |  | 1.19 (0.08-18.77) | 0.901 |
| Ascertainment using diagnostics |  |  |  |  |  |  |  |
| Low ROB | 2 | 120 | 1.00 |  | 0.810 | -- | -- |
| Unclear | 12 | 1,017 | 1.34 (0.13-14.22) | 0.810 |  | -- | -- |
| Ruling out other causes |  |  |  |  |  |  |  |
| Low ROB | 2 | 120 | 1.00 |  | 0.810 | -- | -- |
| Unclear | 12 | 1,017 | 1.34 (0.13-14.22) | 0.810 |  | -- | -- |
| Consistency in diagnostic methods |  |  |  |  |  |  |  |
| Low ROB | 12 | 1,017 | 1.00 |  | 0.810 | -- | -- |
| Unclear | 2 | 120 | 0.75 (0.07-7.96) | 0.810 |  | -- | -- |
| Data completeness |  |  |  |  |  |  |  |
| Low ROB | 8 | 368 | 1.00 |  | 0.006 | 1.00 |  |
| Unclear | 6 | 769 | 0.16 (0.04-0.59) | 0.006 |  | 0.16 (0.00-5.37) | 0.308 |
| Sample size |  |  |  |  |  |  |  |
| <100 | 9 | 444 | 1.00 |  | 0.061 | 1.00 |  |
| ≥100 | 5 | 693 | 0.24 (0.05-1.07) | 0.061 |  | 0.88 (0.03-29.23) | 0.941 |

Abbreviations: aOR, adjusted odds ratio; APS, antiphospholipid syndrome; CI, confidence interval; OR, odds ratio; RA, rheumatoid arthritis; ROB, risk of bias; SLE, systemic lupus erythematosus.

^*^Covariates with p-value ≤0.2 in the univariable analysis were included in the multivariable analysis.

^†^Covariates with p-value <0.05 in the multivariable analysis were considered as showing statistically significant evidence for an association with A5R prevalence.

^‡^Mixed population includes controls and cases with RA, mixed status, and no autoimmune disease.

^§^Mixed population includes controls and cases with RA, SLE, and mixed status.

**References:**

1. Mu F, Wang M, Zeng X, Liu L, Wang F. Preconception Non-criteria Antiphospholipid Antibodies and Risk of Subsequent Early Pregnancy Loss: a Retrospective Study. Reproductive Sciences. 31(3):746–53.

2. Roselli D, Bonifacio MA, Barbuti G, Rossiello MR, Ranieri P, Mariggiò MA. Anti-Phosphatidylserine, Anti-Prothrombin, and Anti-Annexin V Autoantibodies in Antiphospholipid Syndrome: A Real-Life Study. Diagnostics (Basel). 13(15):2507.

3. Murad H, Ali B, Twair A, Baghdadi K, Alhalabi M, Abbady AQ. “In House” assays for the quantification of Annexin V and its autoantibodies in patients with recurrent pregnancy loss and in vitro fertilisation failures. Sci Rep. 13(1):22322.

4. Liu X, Zhu L, Liu H, Cai Q, Yun Z, Sun F, et al. Non-criteria antiphospholipid antibodies in antiphospholipid syndrome: Diagnostic value added. Front Immunol. 13:972012.

5. Anunciación-Llunell A, Muñoz C, Roggenbuck D, Frasca S, Pardos-Gea J, Esteve-Valverde E, et al. Differences in Antiphospholipid Antibody Profile between Patients with Obstetric and Thrombotic Antiphospholipid Syndrome. Int J Mol Sci. 23(21):12819.

6. Hu C, Li S, Xie Z, You H, Jiang H, Shi Y, et al. Evaluation of the Diagnostic Value of Non-criteria Antibodies for Antiphospholipid Syndrome Patients in a Chinese Cohort. Front Immunol. 12:741369.

7. Ho WK, Rigano J. Low prevalence of antiannexin A5 antibodies in unprovoked venous thrombosis. Int J Lab Hematol. 43(5):1225–8.

8. Khogeer H, Altahan S, Alrehaily A, Sheikh A, Awartani K, Al-Kaff M, et al. The Diagnostic Value of New Additional Antiphospholipid Antibodies in Antiphospholipid Syndrome. Ann Clin Lab Sci. 51(4):552–6.

9. Volkov I, Seguro L, Leon EP, Kovács L, Roggenbuck D, Schierack P, et al. Profiles of criteria and non-criteria anti-phospholipid autoantibodies are associated with clinical phenotypes of the antiphospholipid syndrome. Autoimmunity Highlights. 11(1):8.

10. Ferreira TG, Delhommeau F, Johanet C, Gerotziafas G, Bornes M, Cohen J, et al. Annexin-A5 resistance and non-criteria antibodies for the diagnosis of seronegative  antiphospholipid syndrome. Clin Rheumatol. 2020 Apr;39(4):1167–71.

11. Gašperšič N, Zaletel M, Kobal J, Žigon P, Čučnik S, Šemrl SS, et al. Stroke and antiphospholipid syndrome-antiphospholipid antibodies are a risk factor  for an ischemic cerebrovascular event. Clin Rheumatol. 2019 Feb;38(2):379–84.

12. Park HS, Gu JY, Jung HS, Kim HK. Thrombotic Risk of Non-Criteria Anti-Phospholipid Antibodies Measured by Line  Immunoassay: Superiority of Anti-Phosphatidylserine and Anti-Phosphatidic Acid Antibodies. Clin Lab. 2019 Mar;65(3).

13. Rigano J, Ho W. The prevalence of criteria and noncriteria antiphospholipid antibodies in patients with unprovoked venous thromboembolism. Res Pract Thromb Haemost. 2019;3((Supplement 1)):683.

14. Thaler MA, Bietenbeck A, Steigerwald U, Büttner T, Schierack P, Lindhoff-Last E, et al. Evaluation of the sensitivity and specificity of a novel line immunoassay for the  detection of criteria and non-criteria antiphospholipid antibodies in comparison to established ELISAs. PLoS One. 2019;14(7):e0220033.

15. Bećarević M, Sarić M, Stojanovich L, Mirković D, Dopsaj V, Ignjatović S. Anti-annexin A5 antibodies and 25-hydroxy-cholecalciferol in female patients with  primary antiphospholipid syndrome. Clin Rheumatol. 2018 Dec;37(12):3359–64.

16. Akinshina S, Makatsariya A, Bitsadze V, Khizroeva J, Khamani N. Thromboprophylaxis in pregnant women with thrombophilia and a history of thrombosis. J Perinat Med. 2018 Oct;46(8):893–9.

17. Pelusa HF, Pezzarini E, Basiglio CL, Musuruana J, Bearzotti M, Svetaz MJ, et al. Antiphospholipid and antioangiogenic activity in females with recurrent miscarriage  and antiphospholipid syndrome. Ann Clin Biochem. 2017 Sep;54(5):577–83.

18. Carmel-Neiderman NN, Tanne D, Goren I, Rotman-Pikielny P, Levy Y. Classical and additional antiphospholipid antibodies in blood samples of ischemic stroke patients and healthy controls. Immunol Res. 2017 Apr;65(2):470–6.

19. Zhang S, Wu Z, Li J, Wen X, Li L, Zhang W, et al. Evaluation of the clinical relevance of anti-annexin-A5 antibodies in Chinese patients with antiphospholipid syndrome. Clin Rheumatol. 2017 Feb;36(2):407–12.

20. Mekinian A, Bourrienne MC, Carbillon L, Benbara A, Noemie A, Chollet-Martin S, et al. Non-conventional antiphospholipid antibodies in patients with clinical obstetrical APS: Prevalence and treatment efficacy in pregnancies. Semin Arthritis Rheum. 2016 Oct;46(2):232–7.

21. Avriel A, Fleischer S, Friger M, Shovman O, Neuman G, Shoenfeld Y, et al. Prediction of Antiphospholipid syndrome using Annexin A5 competition assay in  patients with SLE. Clin Rheumatol. 2016 Dec;35(12):2933–8.

22. Bećarević M, Stojanović L, Ignjatović S, Dopsaj V. The IgM isotype of anti-annexin A5 antibodies and multiple positivity of  conventional antiphospholipid antibodies: increasing the number of clinical manifestations of primary antiphospholipid syndrome. Clin Rheumatol. 2016 May;35(5):1361–5.

23. Khizroeva J, Makatsariya N, Stuleva N, Abramyan R. Apa profile in women with fetal loss syndrome. International Journal of Gynecology and Obstetrics. 2015;131((SUPPL. 5)):E461.

24. Maneyro A, Ouvina S, Noya L, Palmer L. Anti-annexin V antibodies in patients with obstetric morbility. Journal of Thrombosis and Haemostasis. 2015;13((SUPPL. 2)):280.

25. Conti F, Capozzi A, Truglia S, Lococo E, Longo A, Misasi R, et al. The mosaic of “seronegative” antiphospholipid syndrome. J Immunol Res. 2014;2014:389601.

26. Skare TL, Borba EA, Nisihara R, Utiyama SRR. Anti-annexin 5 in patients with systemic lupus erythematosus. Vol. 32, Clinical and experimental rheumatology. Italy; 2014. p. 448–9.

27. Malickova K, Janatkova I, Zima T. Phosphatidylserine-dependent antiprothrombin antibodies and antibodies towards annexin v play a role in recurrent miscarriages. Biochim Clin. 37(((SUPPL.1)):S101.

28. Pelusa F, Bearzotti M, Svetaz M, Daniele S, Almara A, Bottai H, et al. Antiphospholipid and antioangiogenic activity in women with recurrent abortion and autoimmune diseases. Lupus. 2013;22((1)):100–1.

29. Singh NK, Yadav DP, Gupta A, Singh U, Godara M. Role of anti-annexin A5 in pathogenesis of hypercoagulable state in patients with antiphospholipid syndrome. Int J Rheum Dis. 2013 Jun;16(3):325–30.

30. Conti F, Alessandri C, Sorice M, Capozzi A, Longo A, Garofalo T, et al. Thin-layer chromatography immunostaining in detecting anti-phospholipid antibodies in seronegative anti-phospholipid syndrome. Clin Exp Immunol. 2012 Mar;167(3):429–37.

31. Wolgast L, Wu X, Arslan A, Rand J. From phenomenology to mechanism: Resistance to annexin a5 anticoagulant activity identifies a subset of patients with antiphospholipid antibodies and thrombosis. Blood. 2012;120((21)).

32. Akhter E, Fang H, Wu X, Rand J, Petri M. Annexin a5 resistance identifies a subset of thrombosis patients in systemic lupus erythematosus. Arthritis Rheum. 2012;64((SUPPL. 10))::S742.

33. Lee KO, Kim WJ, Na SJ, Heo JH, Lee KY. Clinical significance of anti-annexin V antibody in acute cerebral ischemia. J Neurol Sci. 2011 Jun;305(1–2):53–6.

34. Tang A, Alfirevic Z, Farquharson R, Dawood F, Quenby. New tests for antiphospholipid syndrome in pregnancy loss. BJOG. 2011;118((8)):1016.

35. Hunt BJ, Wu XX, de Laat B, Arslan AA, Stuart-Smith S, Rand JH. Resistance to annexin A5 anticoagulant activity in women with histories for obstetric antiphospholipid syndrome. Am J Obstet Gynecol. 2011 Nov;205(5):485.e17-23.

36. Rezk A, Abdel-Hafeez N, Rageh I, Abdalla W. Anti-annexin as a marker in patients with recurrent miscarriages. Middle East Fertil Soc J. 2010;15((1)):47–50.

37. Garcia Ruiz I, Zarzoso C, Ferrer-Oliveras R, Lopera AF, Farran-Codina I, Rodrigo-Anoro M, et al. Lack of anti-annexin A5 antibodies in women with spontaneous pregnancy loss. Lupus. 2010;19((4)):532.

38. Wolgast L, Wu X, Niakan J, Arslan A, Rand J. A functional assay for annexin A5 resistance identifies a thrombogenic mechanism in a subset of patients with antiphospholipid antibodies. Blood. 2010;116((21)).

39. Becarevic M, Singh S, Majkic-Singh N. Oxidized LDL, anti-oxidized LDL and anti-annexin A5 antibodies in primary antiphospholipid syndrome. Clin Lab. 2008;54(3–4):97–101.

40. Tomer A, Bar-Lev S, Fleisher S, Shenkman B, Friger M, Abu-Shakra M. Antiphospholipid antibody syndrome: the flow cytometric annexin A5 competition assay  as a diagnostic tool. Br J Haematol. 2007 Oct;139(1):113–20.

41. Mtiraoui N, Zammiti W, Fekih M, Hider S, Almawi WY, Mahjoub T. Lupus anticoagulant and antibodies to beta 2-glycoprotein I, annexin V, and  cardiolipin as a cause of recurrent spontaneous abortion. Fertil Steril. 2007 Nov;88(5):1458–61.

42. Peluso G, Morrone G. [Antiphospholipid antibodies and recurrent abortions: possible pathogenetic role of  annexin A5 investigated by confocal microscopy]. Minerva Ginecol. 2007 Jun;59(3):223–9.

43. Rand JH, Arslan AA, Wu XX, Wein R, Mulholland J, Shah M, et al. Reduction of circulating annexin A5 levels and resistance to annexin A5 anticoagulant activity in women with recurrent spontaneous pregnancy losses. Am J Obstet Gynecol. 2006 Jan;194(1):182–8.

44. Wu XX, Arslan AA, Wein R, Reutelingsperger CP, Lockwood CJ, Kuczynski E, et al. Analysis of circulating annexin A5 parameters during pregnancy: absence of  differences between women with recurrent spontaneous pregnancy losses and controls. Am J Obstet Gynecol. 2006 Oct;195(4):971–8.

45. Zammiti W, Mtiraoui N, Hidar S, Fekih M, Almawi WY, Mahjoub T. Antibodies to beta2-glycoprotein I and annexin V in women with early and late idiopathic recurrent spontaneous abortions. Arch Gynecol Obstet. 2006 Aug;274(5):261–5.

46. Ulcová-Gallová Z, Mukengnábl P, Hadravská S, Bibková K, Slechtová J, Kyselová V, et al. [Placenta and annexin V receptors, antibodies against annexin V and against other  phospholipids in patients with recurrent pregnancy loss]. Ceska Gynekol. 2006 Dec;71(6):469–73.

47. Bizzaro N, Tonutti E, Villalta D, Tampoia M, Tozzoli R. Prevalence and clinical correlation of anti-phospholipid-binding protein antibodies  in anticardiolipin-negative patients with systemic lupus erythematosus and women with unexplained recurrent miscarriages. Arch Pathol Lab Med. 2005 Jan;129(1):61–8.

48. Ishikura K, Wada H, Kamikura Y, Hattori K, Fukuzawa T, Yamada N, et al. High prevalence of anti-prothrombin antibody in patients with deep vein thrombosis. Am J Hematol. 2004 Aug;76(4):338–42.

49. Gaspersic N, Rot U, Cucnik S, Bozic B, Kveder T, Rozman B. Low prevalence of antiphospholipid antibodies in a series of young patients with  cerebrovascular disease. Vol. 21, Clinical and experimental rheumatology. Italy; 2003. p. 680.

50. Roldán V, Marín F, Pineda J, Marco P, Corral J, Climent V, et al. [Annexin V levels in survivors of early myocardial infarction]. Rev Esp Cardiol. 2002 Dec;55(12):1230–4.

51. Nojima J, Kuratsune H, Suehisa E, Futsukaichi Y, Yamanishi H, Machii T, et al. Association between the prevalence of antibodies to beta(2)-glycoprotein I,  prothrombin, protein C, protein S, and annexin V in patients with systemic lupus erythematosus and thrombotic and thrombocytopenic complications. Clin Chem. 2001 Jun;47(6):1008–15.

52. Arnold J, Holmes Z, Pickering W, Farmer C, Regan L, Cohen H. Anti-beta 2 glycoprotein 1 and anti-annexin V antibodies in women with recurrent  miscarriage. Br J Haematol. 2001 Jun;113(4):911–4.

53. Gris JC, Quere I, Sanmarco M, Boutiere B, Mercier E, Amiral J, et al. Antiphospholipid and antiprotein syndromes in non-thrombotic, non-autoimmune women with unexplained recurrent primary early foetal loss. The Nimes Obstetricians and Haematologists Study--NOHA. Thromb Haemost. 2000 Aug;84(2):228–36.

54. Ogawa H, Zhao D, Dlott JS, Cameron GS, Yamazaki M, Hata T, et al. Elevated anti-annexin V antibody levels in antiphospholipid syndrome and their involvement in antiphospholipid antibody specificities. Am J Clin Pathol. 2000 Oct;114(4):619–28.

55. Satoh A, Suzuki K, Takayama E, Kojima K, Hidaka T, Kawakami M, et al. Detection of anti-annexin IV and V antibodies in patients with antiphospholipid syndrome and systemic lupus erythematosus. J Rheumatol. 1999 Aug;26(8):1715–20.

56. Eschwège V, Peynaud-Debayle E, Wolf M, Amiral J, Vissac AM, Bridey F, et al. Prevalence of antiphospholipid-related antibodies in unselected patients with  history of venous thrombosis. Blood Coagul Fibrinolysis. 1998 Jul;9(5):429–34.

57. Kaburaki J, Kuwana M, Yamamoto M, Kawai S, Ikeda Y. Clinical significance of anti-annexin V antibodies in patients with systemic lupus  erythematosus. Am J Hematol. 1997 Mar;54(3):209–13.

58. Kaburaki J, Ikeda Y. [Thrombosis in patients with SLE and antiphospholipid syndrome]. Rinsho Ketsueki. 1995 Apr;36(4):320–4.
